# Police‐initiated diversion for youth to prevent future delinquent behavior: a systematic review

**DOI:** 10.4073/csr.2018.5

**Published:** 2018-06-01

**Authors:** David B. Wilson, Iain Brennan, Ajima Olaghere

## Abstract

This Campbell systematic review examines the effects police‐initiated diversion programs on delinquent behavior, compared to traditional system processing. The review summarizes evidence from nineteen high‐quality studies, including 13 randomized controlled trials and six quasi‐experimental studies.

This review includes studies that evaluated the effects of police‐led diversionary practices compared to traditional processing for youth under 18 years of age. We identified a total of 14 manuscripts representing 19 evaluations. Of these 19 evaluations, 13 used randomized controlled designs (random assignment to conditions) and 6 used quasi‐experimental designs (no random assignment to conditions). Many of these designs included two or more diversionary conditions compared to a common control (traditional processing) producing 31 treatment‐comparison contrasts for analysis. These studies were conducted between 1973 and 2011, inclusively. Most were conducted in the USA (11) with the remaining conducted in Canada (4), Australia (2), and the UK (2).

The general pattern of evidence is positive, suggesting that police‐led diversion reduces the future delinquent behavior of low‐risk youth relative to traditional processing. Assuming a 50 percent reoffending rate for the traditional processing condition, the results suggest a reoffending rate of roughly 44 percent for the diverted youth. This overall benefit of diversion holds for the random assignment studies judged to be free from any obvious risks of bias. No meaningful differences were found across types of diversionary programs. Furthermore, we found no evidence to suggest these findings suffer from publication selection bias.

**Plain language summary:**

**Police‐led diversion of low‐risk youth reduces their future contact with the justice system**

Police‐led diversion of low‐risk youth who come into contact with the justice system is more effective in reducing a youth's future contact with the justice system compared to traditional processing.

**What is this review about?**

Youth misconduct and misbehavior is a normal part of adolescence and that misbehavior sometimes crosses the line from disruptive or problematic to delinquent. Nationally representative surveys of youth in the USA have indicated that minor delinquent behavior is normative, particularly for boys. The normative nature of minor delinquent behavior raises the question of how police should respond to minor delinquent behavior in a way that is corrective, but also avoids involving the youth in the criminal justice system beyond what will be effective in reducing future misbehavior.

Police diversion schemes are a collection of strategies police can apply as an alternative to court processing of youth. Diversion as an option is popular among law enforcement officers, as it provides an option between ignoring youth engaged in minor wrongdoing and formally charging such youth with a crime. Police‐led diversion has the potential to reduce reoffending by limiting the exposure of low‐risk youth to potentially harmful effects of engagement with the criminal justice system.

**What is the aim of this review?**

This Campbell systematic review examines the effects police‐initiated diversion programs on delinquent behavior, compared to traditional system processing. The review summarizes evidence from nineteen high‐quality studies, including 13 randomized controlled trials and six quasi‐experimental studies. This review examined whether police‐led diversion and traditional processing of youth have different effects on rates of official delinquency.

**What are the main findings of this review?**

This review includes studies that evaluated the effects of police‐led diversionary practices compared to traditional processing for youth under 18 years of age. We identified a total of 14 manuscripts representing 19 evaluations. Of these 19 evaluations, 13 used randomized controlled designs (random assignment to conditions) and 6 used quasi‐experimental designs (no random assignment to conditions). Many of these designs included two or more diversionary conditions compared to a common control (traditional processing) producing 31 treatment‐comparison contrasts for analysis. These studies were conducted between 1973 and 2011, inclusively. Most were conducted in the USA (11) with the remaining conducted in Canada (4), Australia (2), and the UK (2).

The general pattern of evidence is positive, suggesting that police‐led diversion reduces the future delinquent behavior of low‐risk youth relative to traditional processing. Assuming a 50 percent reoffending rate for the traditional processing condition, the results suggest a reoffending rate of roughly 44 percent for the diverted youth. This overall benefit of diversion holds for the random assignment studies judged to be free from any obvious risks of bias. No meaningful differences were found across types of diversionary programs. Furthermore, we found no evidence to suggest these findings suffer from publication selection bias.

**What do the findings of this review mean?**

The findings from this systematic review support the use of police‐led diversion for low‐risk youth with limited or no prior involvement with the juvenile justice system. Thus, police departments and policy‐makers should consider diversionary programs as part of the mix of solutions for addressing youth crime.

Many of the studies included in the review were conducted in the 1970s and 1980s. Newer high quality studies are needed to ensure that the findings still hold for contemporary juvenile justice contexts. Additional studies are also needed outside of the USA for this same reason. Finally, we recommend that research explore the usefulness of diversion for low‐risk adult offenders.

**How up‐to‐date is this review?**

The search for eligible studies was completed in January of 2017, so only studies identifiable through January 2017 were included. This Campbell systematic review was published in May 2018.

**Executive summary/Abstract:**

**Background**

Overly punitive responses to youth misconduct may have the unintended consequence of increasing the likelihood of future delinquency; yet, overly lenient responses may fail to serve as a corrective for the misbehavior. Police diversion schemes are a collection of strategies police can apply as an alternative to court processing of youth. Police‐initiated diversion schemes aim to reduce reoffending by steering youth away from deeper penetration into the criminal justice system and by providing an alternative intervention that can help youth address psychosocial development or other needs that contribute to their problem behavior.

**Objectives**

The objective of this review was to synthesize the evidence on the effectiveness of pre‐court interventions involving police warning or counseling and release, and cautioning schemes in reducing delinquent behavior.

**Search methods**

A combination of 26 databases and websites were searched. References of relevant reviews were also scanned to identify studies. We also consulted with experts in the field. Searches were executed by two reviewers and conducted between August 2016 and January 2017.

**Selection criteria**

Only experimental and quasi‐experimental designs were eligible for this review. All quasi‐experimental designs must have had a comparison group similar to the police diversion intervention group with respect to demographic characteristics and prior involvement in delinquent behavior (i.e., at similar risk for future delinquent behavior). Additionally, studies must have included youth participants between 12 and 17 years of age who either underwent traditional system processing or were diverted from court processing through a police‐led diversion program. Studies were also eligible if delinquency‐related outcomes, including official and non‐official (self‐report or third‐party reporting) measures of delinquency were reported.

**Data collection and analysis**

This study used meta‐analysis to synthesize results across studies. This method involved systematic coding of study features and conversion of study findings into effect sizes reflecting the direction and magnitude of any police‐led diversion effect. There were 19 independent evaluations across the 14 primary documents coded for this review. From this, we coded 67 effect sizes of delinquent behavior post diversion across 31 diversion‐traditional processing comparisons. We analyzed these comparisons using two approaches. The first approach selected a single effect size per comparison based on a decision rule and the second used all 67 effect sizes, nesting these within comparison condition and evaluation design.

**Results**

The general pattern of evidence is positive, suggesting that police‐led diversion modestly reduces future delinquent behavior of low‐risk youth relative to traditional processing.

**Authors' conclusions**

The findings from this systematic review support the use of police‐led diversion for low‐risk youth with limited or no prior involvement with the juvenile justice system. Thus, police departments and policy‐makers should consider diversionary programs as part of the mix of solutions for addressing youth crime.

## Background

### The problem, condition or issue

Misbehavior is a normal part of adolescence and that misbehavior sometimes crosses the line from disruptive or problematic to delinquent. Nationally representative surveys of youth in the United States have indicated that minor delinquent behavior is normative, particularly for boys (Bongers et al., 2003; Elliott et al., 1983; Moffitt, 1994). The normative nature of minor delinquent behavior raises the question of how police should respond to minor delinquent behavior in a way that is corrective, but also avoids involving the youth in the criminal justice system beyond what will be effective in reducing future misbehavior. Stated differently, what is the right level of response to minor delinquent acts? Overly punitive responses may have the unintended consequence of increasing the likelihood of future delinquency; overly lenient responses may fail to serve as a corrective for the misbehavior. Police diversion schemes are a collection of strategies police can apply as an alternative to court processing of youth. Police‐initiated diversion schemes aim to reduce reoffending by steering youth away from deeper penetration into the criminal justice system and by providing an alternative intervention that can help youth address psychosocial development or other needs that contribute to their problem behavior.

Diversion as an option is popular among law enforcement officers, as it provides an option between ignoring youth engaged in minor wrongdoing and formally charging such youth. In 2016/17, approximately 60% of first‐arrest juveniles in England and Wales received a caution rather than court‐processing (Ministry of Justice, 2017) and an unknown number were diverted prior to any cautioning. Diversion has the potential to reduce reoffending by limiting the exposure of low‐risk youth to potentially harmful deviant peers within the criminal justice system. Furthermore, diversion may reduce criminal justice system costs, freeing these resources for higher risk youth. However, some commentators (Ray & Childs, 2015; Mears et al., 2016) have noted that diversion may widen the population of youth under the surveillance of the criminal justice system if youth are subsequently punished for failing to meet the terms of their diversion. Consequently, diversion may inadvertently increase youth reoffending. The uncertain potential for diversion to produce both benefits and harms and law enforcement's sustained use of diversion underscores the importance of comprehensively reviewing the effectiveness of these interventions.

### The intervention

This review will focus on the pre‐charge diversion of youth. Police‐led juvenile diversion is a pre‐court intervention initiated by police that represents an alternative to court processing or the imposition of formal charges. Examples might involve a caution, a restorative caution, or a final warning or reprimand. Each of these alternatives might be combined with an additional program element such as referral to a treatment service provider. Police‐led diversions may be known by many names, such as cautions, final warnings, police‐led intervention, police control of juveniles, police‐led proactive prevention, police‐led diversion, pre‐charge diversion or simply as diversion. The essential feature involves police initiating and leading the intervention and the youthful offender receiving a diversionary scheme to avoid a criminal record and any negative consequences that may result from continued formal contact with the criminal justice system (e.g., imposition of formal charges, conviction, etc.).

The participants in a traditional police cautioning scheme include a police officer, the youth in question, and the parents, at a minimum. Victims are not involved nor do police officers routinely receive any training, but solely provide an explanation about the legal and social consequences of continued delinquent behavior. However, variants of this scheme, caution plus and restorative cautioning, involve other interventions and services (Audit Commission, 1996) or involvement of a script with certain questions to structure discussion between an offender and the affected parties and the presence of the victim, in the case of restorative cautioning or conferencing (Wilcox et al., 2004). As for the final warning and reprimand scheme, this involves an assessment‐based approach to evaluate the seriousness of the offense and, depending on the gravity of the offense, a reprimand or final warning with referral to a multi‐agency team for further assessment and placement in a behavioral treatment program (Holdaway, 2003, p. 352).

### How the intervention might work

Wilson and Hoge (2015) articulate two theoretical supports for diversion: labeling theory and differential association theory. Labeling theory posits that the stigmatizing effect of labeling a youth as delinquent may establish expectations for future delinquent acts and alter that youth's social networks toward more deviant peers, thus increasing the likelihood of future deviant behavior (Bernburg et al., 2006). Thus, diverting a low‐risk youth not already labeled as “delinquent” may reduce future offending. Sutherland's (1939) differential association theory states a youth learns the values, attitudes, and techniques of criminal behavior through the interaction with delinquent peers. As such, diverting low‐risk youth from the juvenile justice system may reduce exposure to deviant peers. In essence, a youth who has engaged in a delinquent act may learn more serious forms of delinquency from others already in the juvenile justice system, including the values and attitudes that support involvement in delinquency.

The reintegrative shaming aspect of restorative justice serves as another theoretical mechanism that might explain a positive effect of diversion. Reintegrative shaming emphasizes an intervention strategy where responses to transgressions are de‐stigmatizing and inclusive of “a meaningful community‐based process that reaffirms the boundaries of acceptable behavior” (Zhang, 2011, p. 2325). These responses should aim to reduce or inhibit new or further stigmatization as a result of contact with the justice system. Forgiveness and non‐stigmatization are central principles of reintegrative shaming, as these tenets reinforce another core feature—reintegration. Reintegration concerns efforts to restore offenders (and victims alike) after a transgression and reintegrate them back into the community. This process also implicates communities of care such as significant others, e.g., family members or individuals of import in an offender's life who are central to disavowing unlawful behavior and facilitating forgiveness. Our interpretation of community of care includes that of authority figures such as police officers, whom we understand may not traditionally be viewed as members of an affected person's broader prosocial community. Yet, the interaction between the police officer and the youth provides an opportunity for an authority figure to reinforce appropriate norms by briefly detaining the youth engaged in a problem behavior, thus enacting an element of shame. The shame may be enhanced by the police officer taking the youth to her home and discussing the youth's acts with her parent. Additionally, the element of reintegration emerges with diverting the youth from any further court processing, allowing the youth to return to a state of “good standing” in the community (Hay, 2001; Sherman, 1993).

Referral to needed and effective services remains as a final mechanism that might contribute to the effectiveness of some diversion schemes. As discussed above, some diversion programs involve formal referrals to treatment services or a needs assessment for services. As the first point of contact with the justice system, police officers are poised to intervene early and provide referrals to needed services that may be more beneficial in reducing future delinquent acts than court processing (Butts, 2016; Mears et al., 2016). Diversionary practices, however, may also be harmful in the sense of increasing a youth's propensity to engage in delinquent behaviors. From a deterrence perspective, diversion provides a swift sanction, but the sanction may be too mild to deter a youth from similar (or more severe) behavior in the future. A youth who perceives that he was not held responsible for his actions may think that he “got away with it” and will continue to engage in similar ways.

Mears et al. (2016) also suggest a possible “net widening” effect of diversion programs. When a diversion with conditions is used instead of a diversion with ‘no further action’ and a youth fails to meet the specified conditions, such as attending an appointment with a counselor, the youth may be brought into the criminal justice system as a consequence. Perversely, this may result in a low‐risk youth experiencing the negative consequences of juvenile justice system involvement that the diversion was designed to prevent.

### Prior reviews

Several meta‐analyses of diversion programs exist and these differ from each other and from the proposed review in important ways. In a Campbell Collaboration review, Petrosino et al. (2010) examined the effectiveness of juvenile justice system processing compared to any alternative non‐system condition. Their focus was on any form of diversion, whereas our focus is on pre‐charge or police‐initiated diversion only. Petrosino et al. (2010) found that court processing produced worse outcomes than diversion from the system. Similarly, Wilson and Hoge (2015) examined 45 studies and found that, on average, diversion conditions had lower recidivism rates than formal court processing. Additionally, their analysis showed slightly larger beneficial effects for pre‐charge diversion compared to post‐charge diversion. However, methodologically stronger research designs failed to find a positive effect for diversion relative to traditional processing. Finally, a meta‐analysis completed by Schwalbe et al. (2012) focused on diversion programs with a treatment component such as case management, family treatment, youth court, etc. Although not explicitly stated, these diversion programs likely occurred post‐charge. The findings showed a small overall effect favoring these programs, but the effects were not statistically significant except for family treatment. Overall, Schwalbe et al. (2012) did not provide a meaningful examination of police‐led diversions, which are the focus of the current review.

Taken as a whole, these meta‐analyses provide an equivocal answer regarding the effectiveness of diversion programs. Furthermore, these prior reviews did not specifically focus on pre‐charge or police‐led diversion; we aim to clearly describe the effect of pre‐charge diversion on reoffending outcomes compared to court processing. Based on labeling and differential association theories, we would expect diversion at this stage to be more effective as it avoids any labeling of the youth, even if temporarily via a formal charge and at a minimum, reduces potential exposure to deviant peers in the juvenile justice system. Hence, the purest form of diversion occurs at this stage per the avoidance of any juvenile justice system processing.

Despite the uncertainty regarding the effectiveness of diversionary practices, diversion schemes are used frequently by police officers. Precise estimates of the prevalence of diversion are difficult given that a central feature of much diversion involves no court processing or recording in criminal justice records. Consequently, prevalence estimates of police‐led youth diversions are rare or must be extrapolated to national levels from small area studies. Additionally, definitions of diversion vary by jurisdiction, further impeding accurate estimates of prevalence. For example, the option of a ‘caution’ that exists in England and Wales, South Africa and Australia still forms part of a youth's juvenile record, but is not a formal charge. Therefore, it is debatable whether these constitute a diversion as discussed above. It is also important to note that police‐led juvenile diversion is not a disposal option in some jurisdictions. For example, Algeria, Argentina, Italy and Kuwait all forbid police‐led diversion, although options for diversion later in the judicial process may exist (Hazel, 2008). According to a Ministry of Justice study, 18% of youth arrests in England and Wales result in a caution (Ministry of Justice, 2017). In the United States, Puzzanchera and Kang (2008) estimate that a similar number (25%) of youth entering the juvenile justice system are diverted, but much of this is initiated post‐charge, rather than by the police officer during the initial interaction with the youth.

Departing from, but building on the work of prior reviews, our systematic review and meta‐analysis focuses on pre‐charge (police‐led) diversion prior to the imposition of formal charges. This narrower focus will help inform police practice related to the use of diversion. Furthermore, we explore the differential effectiveness of the various diversionary schemes, such as a diversion with no further action, restorative caution, or diversion with various therapeutic elements.

## Objectives

### The problem, condition or issue

The objective of this review was to synthesize the evidence on the effectiveness of pre‐court interventions involving police warning, reprimand, and cautioning schemes in reducing delinquent behavior. Our specific research questions were:

1. Are police‐initiated diversions effective in reducing future delinquent behavior (i.e., additional cautioning, arrest, court appearances, or findings of guilt)?
2. Is effectiveness related to the type of police‐initiated diversion used (i.e., traditional cautioning, caution plus, police restorative cautioning, final warning or reprimand)?
3. Is effectiveness related to characteristics of the youth (i.e., age, gender, race/ethnicity, crime committed, and offense history)?

## Methods

### Deviations from protocol

This review has deviated from the published protocol in a few ways. These deviations are listed and explained below and do not impact the results of the review.

- We originally proposed to examine the impact of police‐initiated diversion on secondary outcomes such as perceptions of law enforcement, perceived fairness, and satisfaction. We were unable to identify enough of these secondary outcomes in the literature to undertake a meaningful review.
- Our *a priori* planned moderator analyses for diversion type originally specified interventions such as traditional cautioning, caution plus, police restorative cautioning, and final warning or reprimand. These categories were collapsed to traditional cautioning, caution plus with a service referral, and police restorative cautioning and inform the moderator analysis for diversion type as shown in table 4.

### Criteria for considering studies for this review

#### Types of studies

Both experimental and quasi‐experimental designs were included. The specific eligibility criteria for each design is detailed below. Qualitative studies were not eligible for inclusion in this review.

- Experimental designs. Eligible experimental designs must have randomly assigned participants to a diversion or a control condition(s). Designs that used a quasi‐random assignment procedure, e.g., assignment based on an alternate case basis, were also eligible.
- Quasi‐experimental designs. Several types of quasi‐experimental designs were eligible; however, all quasi‐experimental designs must have had a comparison group that was similar to the police diversion intervention group with respect to demographic characteristics and prior involvement in delinquent behavior (i.e., similar risk for future delinquent behavior). This similarity can be achieved through matching or statistical controls. Matching at the individual level or at the group level was permitted. Statistical control methods could include regression analysis, analysis‐of‐covariance, and propensity score modeling, among others. Use of a statistical control method was sufficient for inclusion meaning, we did not exclude studies based on a subjective assessment of the quality of the statistical controls. Rather, any quasi‐experimental design that controlled for baseline risk factors, such as age, gender, and prior offense history, was eligible. Quasi‐experimental designs were not eligible if the comparison group was comprised of participants who refused participation in a police diversion scheme or who dropped out of a police diversion scheme. Quasi‐experimental designs that did not have a comparison group were not eligible.

#### Types of participants

The population of interest was youth suspected of involvement in a crime or delinquent behavior. Eligible studies must have included participants who were youth between 12 and 17 years of age, inclusively. Participant samples that included a small proportion (i.e., less than 20%) of youth over 17 but less than 22 were also eligible. Participants must also have been apprehended, arrested, or otherwise referred to the juvenile justice system, and either diverted to a police‐involved intervention prior to the imposition of formal charges, or in the case of a comparison condition, treated in some other fashion.

#### Types of interventions

Interventions that were considered eligible must have been initiated and implemented by police officers as identified by the study. This included programs where diversion occurred any time prior to formal charges—whether before or after arrest—but prior to the imposition of formal charges. Interventions that involved court or prosecutorial referrals, even with the inclusion of police officers, were not considered eligible. Findings were considered relevant if measured at the police ‘level of referral’. Control conditions were typically ‘treatment as usual’, which is often a process of laying criminal charges followed by adjudication through the criminal justice system. Also excluded were studies where a disposal of ‘no further action’ was treated as a control condition.

#### Types of outcome measures

##### Primary outcomes

The primary outcome of interest was delinquency. Eligible studies must have reported at least one delinquency‐related outcome. This could include official measures of delinquency, such as an arrest, or other measures of delinquent‐type behaviors, such as self‐report, parent‐report, or school records of wrongdoing.

##### Secondary outcomes

Secondary outcomes of interest could have included self‐report measures related to improved relations, such as satisfaction with police or the cautioning process.

### Search methods for identification of studies

#### Electronic searches

Four categories of key words were developed for this search. The first category lists key terms and synonyms related to youth and their social status. The second category of key terms are related to pre‐court cautioning practices and schemes. The third and fourth categories address the methodology and the measured study outcomes, respectively. Zotero, a reference management software program was used to retrieve, store, and document the search process. Each database had its own file folder within Zotero and was searched individually. Search notes were created for each database and stored in the appropriate file folder. The search notes captured: the date of the search, the database name, the final search string used, the reference yield produced, and a notes field to capture any aberrant issues.

##### Population

youth OR child OR juvenile OR delinquent OR devian* OR student OR adolescent OR “young person” OR “young offender*” OR bully* OR “youthful offender”

##### Treatment

diverted OR diversion OR caution* OR “caution plus” OR restorative OR “restorative caution” OR triage OR “final warning” OR reprimand OR “alternative* to custody” OR “pre‐charge” OR “pre‐caution” OR “pre‐court” OR “pre‐custody” OR “alternative program*” OR disposal OR disposition OR liaison OR Police‐led OR “police initiated” OR “police control” OR “police diversion” OR police OR “law enforcement” OR “civil citation”

##### Methodology

outcome OR evaluat* OR effect OR effectiv* OR experiment* OR quasi OR assessment OR RCT OR “random* control*”

##### Outcome

recidivism OR arrest* OR rearrest* OR citation OR offend* OR reoffend* OR conviction OR reconviction OR adjudication OR adjudicated

This search strategy was applied to the following databases and websites, which cover both the easily accessible sources as well as the grey literature.

**Table:** 

Australian Institute of Criminology	PolicyFile
Center for Problem Oriented Policing	ProQuest Criminal Justice
Australian Criminology Database via Informit	Dissertations & Theses: Full Text
Criminal Justice Abstracts	OVID
EconLit	PubMed
First Search—Dissertation Abstracts	PsycINFO
Global Policing Database	ProQuest Dissertations & Theses
Google Scholar	Public Affairs Information Service
HeinOnline	RAND Documents
Home Office (including archives)	Safetylit.org
Ministry of Justice	Social Sciences Citation Index
NCJRS (National Criminal Justice Reference Service)	Social Services Abstracts
Policy Archive	Sociological Abstracts

#### Searching other resources

In addition to searching the electronic resources listed above, we also scanned references of relevant reviews and identified studies. We also consulted with an expert in the field, Peter Neyroud, a lecturer at the Institute of Criminology, University of Cambridge, and received a list of experimental and quasi‐experimental studies.

### Data collection and analysis

#### Data extraction and management

**Figure 1:**
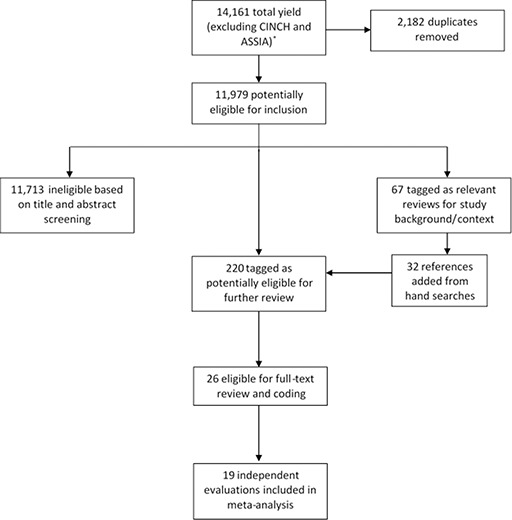
Reference flow diagram

The second and third author screened titles and abstracts for relevance and then equally divided the smaller pool of resulting studies to review the full‐text of the reference for eligibility. At this stage, reviewers also hand searched relevant, but ineligible references—prior meta‐analyses and reviews (see Farrington & Welsh, 2006; Petrosino et al., 2010; Schwalbe et al., 2012; Wilson & Hoge, 2013)—to be exhaustive and cross‐reference for additional references not captured in the systematic search. These references were then sorted into two categories—a relevant reference for further review or a reference relevant for background information or context (see Figure 1). Next, each reviewer applied the inclusion criteria noted above to review the full‐text of the smaller set of studies. From this, the final eligible sample of studies emerged and the second and third author fully coded each study in this final sample. At this stage reviewers also eliminated studies that were coded as eligible, but upon further review and through consensus meetings were deemed ineligible (see Patrick & Marsh, 2005). All three authors were involved in the coding and double coding of the final set of eligible studies. All three authors also reconciled coding differences through weekly consensus meetings.

We coded eligible studies for study characteristics, intervention/comparison characteristics, outcome characteristics, and effect size data (see coding forms in appendix A). Coding was unique for each eligible study, as the unit of analysis for the meta‐analysis is an independent study. In cases where there were multiple publications for the same study, the most complete study was coded as the primary study and all other related publications were coded as cross‐references. Included studies were double‐coded with differences resolved through consensus meetings. All coding and data management, including capturing decisions from consensus meetings was managed using FileMaker Pro database software.

#### Assessment of risk of bias in included studies

Methodological quality and risk of bias was coded as data was extracted for study, intervention/comparison, and outcome characteristics. Specifically, at the study level, we coded for the type of experimental and quasi‐experimental design based on assignment (e.g., matching, wait list control, cohort, etc.). Risk of bias was captured by assessing the risk of selective outcome reporting. At the intervention/comparison level, risk of bias was coded based on reported or observed differences between groups at baseline (selection bias) and attrition bias for the primary outcome, in terms of quantity and differential attrition. Finally, at the outcome level risk of bias was based on one item, which captured whether there was potential bias from non‐blinding procedures.

#### Measures of treatment effect

The primary outcome for this review is delinquency and is most often reported on a dichotomous scale, that is, as delinquent or non‐delinquent. As such, the effect size of choice for this review was the odds ratio. Odds ratios were computed from any available information such as proportions, percentages, raw frequencies, chi‐square and marginal distributions, etc. In the case of quasi‐experimental designs with statistical adjustments for baseline differences, the regression coefficient from a logistic regression model was coded as the logged odds ratio along with the reported standard error. Effect sizes based on scaled measures of delinquency were computed as *d*‐type effect sizes and then converted to odds ratios using the logit transformation method (Lipsey and Wilson, 2001). All effect size computations used established equations as implemented in the online effect size calculator available on the Campbell website.

#### Assessment of heterogeneity

In place of the traditional ‐statistic, heterogeneity was assessed using τ^2^, which is the random effects variance component used in random effects models. We did not report *Q* because we used the robust standard errors method of analysis for handling statistically dependent effect sizes (Hedges, Tipton, & Johnson, 2010). This method does not produce a formal test of the statistical significance of τ^2^. When τ^2^ is zero, the distribution is homogeneous. As τ^2^ increases, it indicates increasing levels of heterogeneity. To get a sense of when τ^2^ might be statistically significant, analyses were rerun using David Wilson's *meanes* macro for Stata, ignoring the statistical dependencies issue. These analyses suggested that with these data, any τ^2^ above 0.05 likely reflects statistically significant heterogeneity. This should be treated as a rough guide only and not as a formal indicator of significant heterogeneity.

#### Assessment of reporting biases

Publication‐selection bias was assessed in three ways. First, analyses compared the results from published and unpublished reports. Published documents included peer‐reviewed journal articles, books, and book chapters. All other report forms, such as theses, technical reports, government and agency reports, were considered unpublished. Second, we performed a trim‐and‐fill analysis. Third, we visually inspected a funnel plot.

#### Unit of analysis issues

Studies based on the same sample were treated as a single study, of which the manuscript with the most complete information was coded as the primary study. The other related documents were consulted for additional information and are part of the reference list of included studies. Publications or documents with multiple independent evaluations within were coded as separate studies, such as studies with RCTs conducted in two different cities.

#### Data synthesis

Meta‐analysis was conducted using random effects models. Primary analyses were performed using the robust standard error method of modeling statistical dependences as implemented in the Stata package *robumeta* (see http://www.northwestern.edu/ipr/qcenter/RVE‐meta‐analysis.html for details).

#### Sensitivity analysis

Our a priori planned moderator analyses included the type of diversion (e.g., diversion only, diversion plus services, and diversion plus police‐led restorative cautioning), the type of research design (e.g., experiment versus quasi‐experiment), and publication type (i.e., published versus unpublished). Post hoc moderator analyses explored the relationship between other study features such as data collection period and country of intervention, and effect size. Meta‐analytic regression was used to complete all moderator analyses using the robust standard errors method.

## Results

### Description of studies

#### Results of the search

The search strategy yielded 14,161 references. Removal of duplicates reduced this to 11,979 references. A pre‐eligibility screening of titles and abstracts identified 220 references potentially relevant from this reduced yield. During this process an additional 67 references were also tagged as relevant for background information to help contextualize this review. Two coders examined the 220 potentially relevant documents in detail for eligibility. Fourteen documents representing 19 unique studies satisfied our eligibility criteria. As is often the case, several studies that were initially coded as eligible were later recoded as ineligible upon closer inspection. In the case of the current review, one study met the criterion for being a near‐miss study. This study is listed in the reference section as marginally eligible, but excluded. Appendix B includes a full list of studies included in and excluded from the meta‐analysis.

#### Included studies

Tables 1, 2, and 3 provide descriptive information on 31 treatment‐comparison contrasts detailed across the 19 studies in the 14 publications. As shown in Table 1, evaluation of 10 of the studies was based on technical reports. In several cases, these reports were also published in a peer‐reviewed journal, but the associated technical reports were preferred as they provided more detailed information about the study. Three of the studies were based on a Master's (n=1) or a PhD thesis (n=2). The remaining five studies were each published as a peer‐reviewed journal article (n=4) or a peer‐reviewed agency report (n=1). The publication dates of the studies ranged from 1979 to 2015. Of the 19 studies, 11 were conducted in the United States – of all which were conducted between 1974 and 1997, with the majority taking place in the late 1970s and early 1980s. The four studies that were conducted in Australia took place between 2000 and 2010; one Canadian study took place in the mid‐1970s and one in the mid‐1990s; and the two UK‐based studies were conducted between 2009 and 2011. Most studies were conducted within a single police department area (usually a city; n=15) although four studies covered a wider area such as a county (Koch, 1985) or a region (Cunningham, 2007; Little, 2015).

As shown in Table 2, of the 31 interventions included in the review, 13 examined traditional caution, 14 examined caution with a referral to services, and 4 examined police restorative cautions. Of the 21 studies that included a referral to services, these services were predominantly provided by external agencies (n=20) and, generally were provided by a public or non‐profit agency and were not purchased (the exception was Klein, 1986, although purchase of services may not always have been reported). The control condition was almost always formal court processing, which equates to a “treatment as usual” condition; one study used probation as the control condition.

The age range of juveniles at the point of entering the study was 10 years to 17 years. Although age range was not reported in six studies, the interventions were limited to juveniles, so all participants would have been between the minimum age of criminal responsibility for the study area and 17 years of age. The gender distribution of samples was typical of the juvenile offending population, with all studies that reported this statistic having a predominantly male sample. The ethnic composition of the study samples was not well‐reported with eight studies not including any information about the ethnicity of their samples.

The most common outcome measure was a binary indicator of arrest or police contact, which was used in 23 of the 31 comparisons. Self‐reported delinquency was used in ten comparisons – the majority of times this measure was calculated by summing the different offence types they committed. The severity of self‐reported delinquency was also measured in one study. For studies that included police or court records as the outcome measure, follow‐up periods were typically in excess of 12 months. All studies that used a self‐reported delinquency measure had follow‐up periods less than one year. Within studies, follow‐up time was consistent across participants in all but three studies: one followed participants to a fixed age (19.5 years) while follow‐up periods in two other studies varied across individuals (15‐21 months and 15‐30 months).

All studies compared treatment effectiveness at the individual level and study designs were consistent within studies when there were multiple comparisons. Studies employed either a quasi‐experimental (6) or a randomized controlled design (13). All the quasi‐experimental studies matched individuals or statistically controlled for baseline differences between treatment and control groups. All but one of the randomized controlled studies were undertaken without matching.

### Risk of bias in included studies

Risk‐of‐bias assessment is shown in Table 3. For each potential source of bias, we coded each study on the following scale: low‐risk, high‐risk, and unclear. The dimensions of bias assessed were: (1) selection (were there meaningful differences between groups at baseline?), (2) general attrition (was there a risk of general attrition bias for the primary outcome measure?), (3) differential attrition (was there a risk of meaningful differential attrition bias for the primary outcome measure?), (4) measurement (did the measure used create a risk of bias on the part of the assessor?), and (5) outcome reporting (is there any evidence that the authors have not reported findings for all variables measured as part of this study?).

The internal validity of the studies was generally high. Study designs were used that were capable of ensuring there were few baseline differences between treatment groups. The use of police records as an indicator of reoffending limited the potential for bias in the data collection process and virtually eliminated the potential for differential rates of study attrition across treatment conditions.

However, there were some limitations to the internal validity of the studies. There was no potential for the blinding of treatment condition on the part of the participants. This was a particular concern when the outcome measures were based upon self‐reported delinquency. Theoretically, respondents may have been supportive of a disposal they regarded as lenient and may have biased their self‐reported delinquency accordingly. It is noteworthy that participant blinding in this type of intervention is not possible and not desirable, as awareness of being diverted is part of the intervention. Police officers, who were responsible for generating the arrests upon which the outcome measures were based, were also not blind to the treatment group of an individual. Hypothetically, this could have impacted their use of discretion in the decision to arrest a study participant at some future time point after the initial diversion. However, we saw no evidence of participant or administrator bias in any of the study reports.

At the level of treatment assignment, some studies reported failures in the randomisation process when police officers were responsible for randomisation. For example, in the New York City study completed by Dunford et al (1982), the project team found that police officers were funneling offenders whom they regarded as low‐risk towards the diversion scheme while funneling higher‐risk offenders towards the control condition. Efforts were made to rectify those treatment assignment biases when they were identified and they also undertook baseline comparisons of treatment group characteristics and statistically adjusted for these biases when appropriate.

There was little to no evidence that attrition or loss of study participants over the course of the study was an issue for these studies. We rated only one study as being at risk of general attrition bias and no study as at risk of differential attrition. The latter is relevant for the internal validity of a study, whereas the latter affects the generalizability of any observed findings. What we were not able to assess was the degree to which the selection of youth into each study's sample was representative of all eligible youth.

### Synthesis of results

Across the 19 independent evaluations identified across the 14 primary documents coded for this review, multiple comparisons of diversion versus traditional processing were common across these evaluations. This also included comparisons of diversion with no referral to services (simple counsel and release) and diversion with referral to services compared to a traditional processing condition. We coded both of these comparisons when possible, producing 31 comparisons for analysis (see Table 2 for details).

These 31 comparisons came from 19 independent evaluations and in cases with multiple comparisons from a single evaluation, the comparisons had a common control group. That is, many of the evaluations included two or more diversion conditions compared to a single control condition. As discussed in the methods section, we used the robust standard errors method of analysis to accommodate these statistical dependencies.

We coded 67 effect sizes of delinquent behavior post diversion across the 31 diversion‐traditional processing comparisons. We analyzed these comparisons using two approaches. The first approach selected a single effect size per comparison based on a decision rule and the second used all 67 effect sizes, nesting these within comparison condition and independent evaluation. The decision rule for selecting effect sizes for the primary analysis was: (1) select the official measures of delinquency (i.e., drop self‐report measures), (2) select effect sizes based on dichotomous indicators of delinquency, (3) select effect sizes closest to 12‐months post diversion, (4) give preference to measures based on arrest over court appearances or convictions, (5) give preference to regression‐adjusted effect sizes over raw effects for quasi‐experimental designs. The logic of this decision‐rule was to give preference to the most common outcome source (official measures) and most common outcome type (dichotomous) and the most common time post diversion (12‐months). These first three are designed to increase the comparability of the effect sizes used in the analysis. The fourth rule gives preference for arrest over court appearances or convictions. The latter two represent deeper penetration into the juvenile justice system and as such are more likely to be affected by other factors, including prior actions by the system. Finally, the decision rule gave preference to regression adjusted effect sizes from quasi‐experimental designs as these are adjusted for observed baseline differences between groups and as such are likely be less biased. This produced a single effect size for all but two comparisons (i.e., Little, 2015; Haines et al. 2012, Lewisham evaluation). In these latter two cases, the remaining effect sizes were averaged to produce a single selected effect size per comparison.

The most common method of effect size computation for these selected 31 effect sizes was based on dichotomous outcome data (e.g., 2 by 2 frequency data or proportions/percentages). This was the case for 29 of these 31 effect‐sizes. For two of the treatment‐comparison contrasts (two independent contrasts from the same study), the effect size was based on a study reported Cohen's *d* effect size, converted to a logged odds‐ratio. For the full collection of 67 effect‐sizes, 46 were based on dichotomous outcome data, 16 were based on means and standard deviations, 4 were based on a logistic regression coefficient, 2 were based on hand computations. All of the effect‐sizes from the latter two methods were consistent in magnitude with the general distribution of effects (e.g., were not extreme). A meta‐regression with robust standard errors compared each method to the null category of dichotomous outcomes. The mean effect‐sizes based on means versus dichotomous outcomes were quite similar and not statistically significantly different. The mean for effect‐sizes based on logistic regression models was slightly small (less positive result), but not significantly so. However, the mean for the two effect‐sizes based on hand computations did differ significantly from the null category of dichotomous outcomes. These two effects were in the direction of diversion being less effective than the alternative so leaving these in the model makes our overall results more conservative.

#### Overall synthesis results

The results of the effect size analyses are shown in Table 4. The general pattern of evidence is positive, suggesting that police‐led diversion modestly reduces the future delinquent behavior of low‐risk youth relative to traditional processing. The random effects mean odds ratio across the 31 selected effect sizes and for all 67 effect sizes were roughly the same (mean odds ratio = 0.77 and 0.82, respectively, with 95% confidence intervals of 0.63 – 0.95 and 0.66 – 1.00, respectively). Both of these mean effect sizes are statistically significant at the .05 level. The size of the effect is modest, however. Assuming a 50 percent reoffending rate for the traditional processing condition, the odds ratio of 0.77 indicates a reoffending rate of 44 percent for the diverted youth.

**Figure 2:**
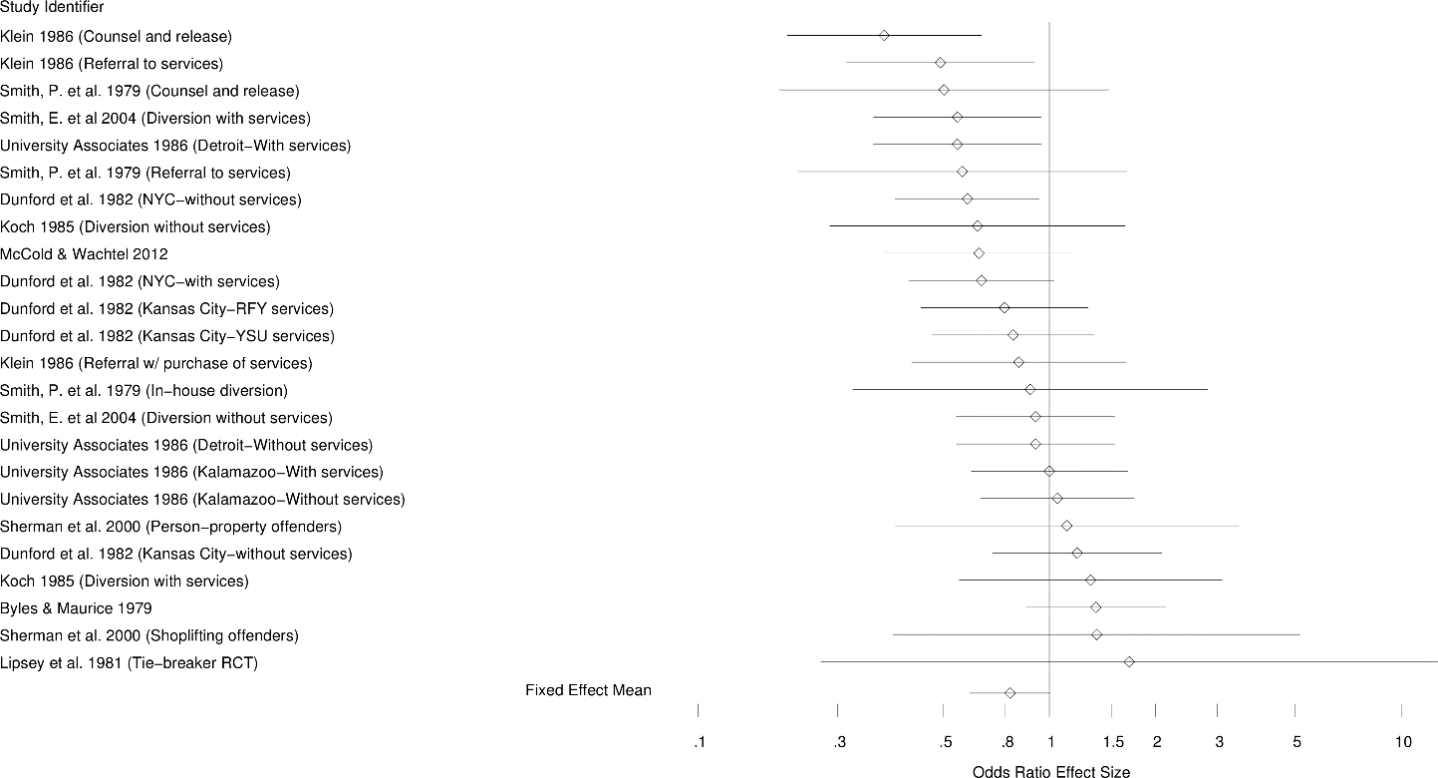
Forest plot for randomized studies

**Figure 3:**
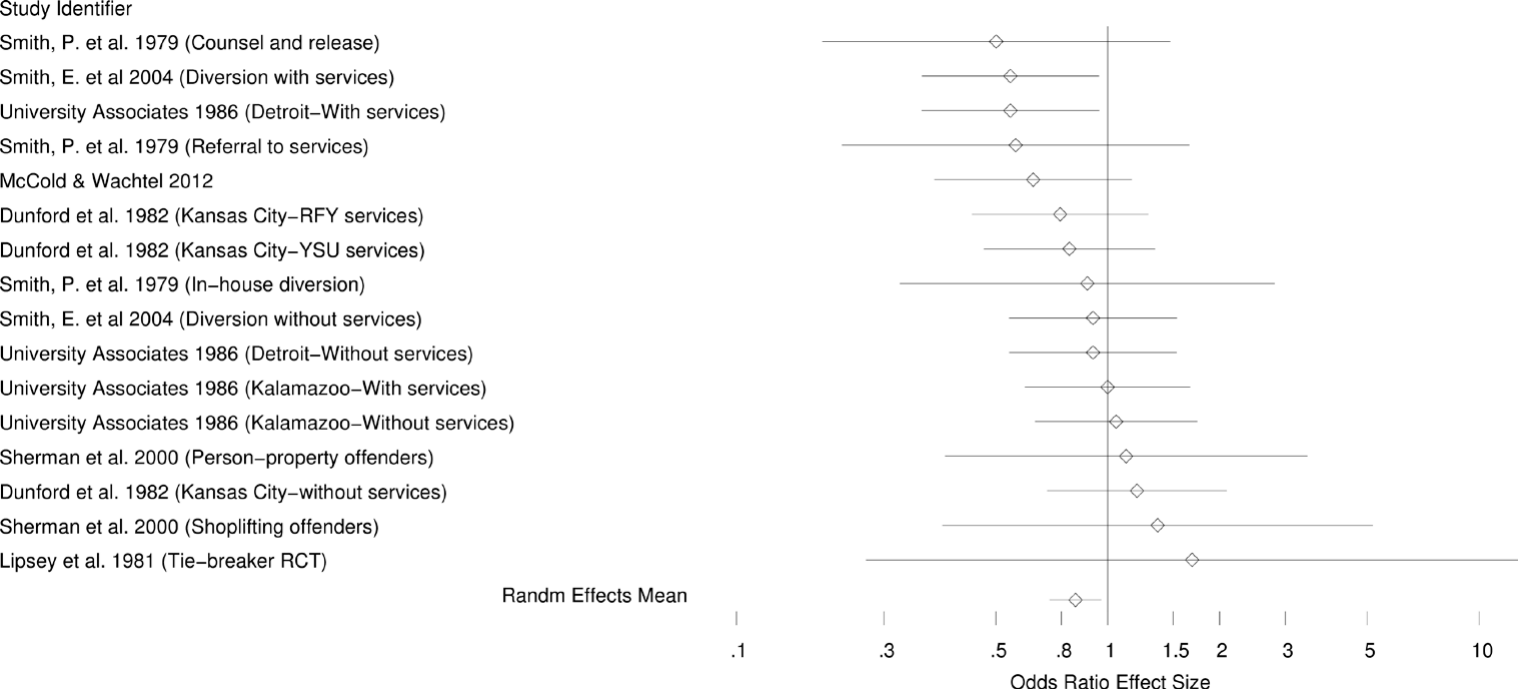
Forest plot for randomzied studies (no indication of bias)

**Figure 4:**
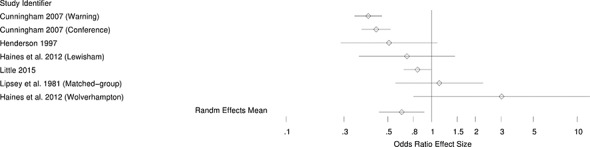
Forest plot for quasi‐experimental studies

Figures 2 – 4 present the forest plots of the selected effects for random assignment studies and quasi‐experimental studies. As can be seen in these figures, the general pattern of effects is to the left of the no‐effect value of one, suggesting generally beneficial effects of diversion.

#### Analysis by risk of bias

The overall analysis ignores research design and risk of bias. To address this, we computed the results separately by design and risk of bias to examine the impact of these factors on the results for the 31 selected effect sizes and all 67 effect sizes (also shown in Table 4). Twenty‐four of the 31 contrasts used a random assignment design and the mean odds ratio for this design was similar to the overall result (mean = 0.82, 95% C.I. = 0.66 – 1.02). In contrast, the effect was larger (farther from the null value of 1) for the quasi‐experimental designs (mean = 0.72, 95% C.I. = 0.40 – 1.27). The results are similar for the analysis based on all 67 studies. These two analyses, however, don't fully address risk of bias as several of the random assignment studies had evidence of randomization failures or meaningful differences between groups at baseline. To account for this, we used our ratings for seventeen of the 24 random assignment contrasts coded as having low attrition (general or differential), low risk of selection bias with no indication of randomization failure or unbalanced groups at baseline. The overall mean for these effects is statistically significant and homogeneous (mean odds ratio = 0.81, 95% C.I. = 0.74 – 0.88, τ^2^ = 0.000). The effects for studies with high risk of bias were roughly the same in most cases, but somewhat larger for studies with an unclear risk of bias. All confidence intervals, however, substantially overlap suggesting that any differences across these categories of risk of bias are not meaningful. Furthermore, we examined 16 comparisons rated as having low risk of bias across all indicators of bias (see Table 3) and observed similar results (mean odds ratio = 0.82, 95% C.I. = 0.68 – 0.98, τ^2^ = 0.000). The results are similar for the analyses based on all effect sizes, although these analyses have more heterogeneity given the greater diversity in outcomes, increasing the confidence interval such that the mean is no longer statistically significant at a conventional level for the lower risk of bias studies. The effects for studies with high risk of bias are roughly the same in most cases, but somewhat larger for studies with an unclear risk of bias. All confidence intervals, however, substantially overlap suggesting that any differences across these categories of risk of bias are not meaningful.

#### Analysis by type of diversion program and country

As shown in Table 1, we categorized the diversion programs into one of four types: (1) diversion only (i.e., caution and release), (2) diversion with referral to services, either internal or external to the criminal justice system, (3) diversion with police‐led restorative justice, and (4) other form of police‐led diversion. Results comparing the first three of these categories are shown in Table 4. We are not showing results for the “other” category, as this would not be meaningful given that only two contrasts from one evaluation were in this category. Furthermore, as the “odd‐ball” category it was not of substantive interest.

Although the results appear to vary across diversion type, these differences are not statistically significant. A meta‐regression model in *robumeta* showed that both the difference between diversion only and diversion with referral to services and the difference between diversion only and diversion with restorative justice were not significant (*t* = 1.41, *p* = 0.180, *t* = 0.08, *p* = 0.939, respectively). This analysis is an indirect assessment of these differential effects given that different studies contributed to each category. However, eight studies compared both diversion only and diversion with referral to services to a common traditional processing condition. A meta‐regression model was run testing the effect of referral to services with these studies (not shown in Table 4). The result was not statistically significant, but the effect modestly favored the referral to services condition, contrary to the difference shown in Table 4 (β = ‐.10, *t* = ‐0.71, *p* = 0.506, τ^2^ = 0.0174). Thus, the current evidence does not suggest any meaningful difference across these variations in diversion type and the small‐observed differences should not be interpreted as suggestive of differential effectiveness.

We also examined whether country of study affected the findings. Given the small number of non‐United States studies, we compared the mean odds ratio for studies conducted in the United States to all studies conducted elsewhere (i.e., Australia, Canada, and the UK). Although the mean odds ratio indicated a modestly large beneficial effect for U.S.‐based studies, the difference was small and not statistically significant (a mean odds ratio of 0.76 versus 0.85, respectively). Thus, the current evidence does not provide a basis for concluding that there is a meaningful difference in diversion effects between these different Anglophone countries.

#### Publication selection bias

We assessed for publication selection bias in three ways, each suggesting that our results are unlikely to be overly influenced by it. First, we compared the results for journal articles relative to other publication or manuscript forms, primarily technical reports and dissertation theses. A full 74 percent of our comparisons came from the latter, in itself a protection against publication selection bias. Table 4 shows the mean odds ratio for journals and for these other manuscript types. Surprisingly, the mean odds ratio for journals (mean = 0.91, 95% C.I. = 0.51 – 1.61) was closer to the null value than the other manuscript types (mean = 0.73, 95% C.I. = 0.58 – 0.92). With publication selection bias we would expect the opposite.

**Figure 5:**
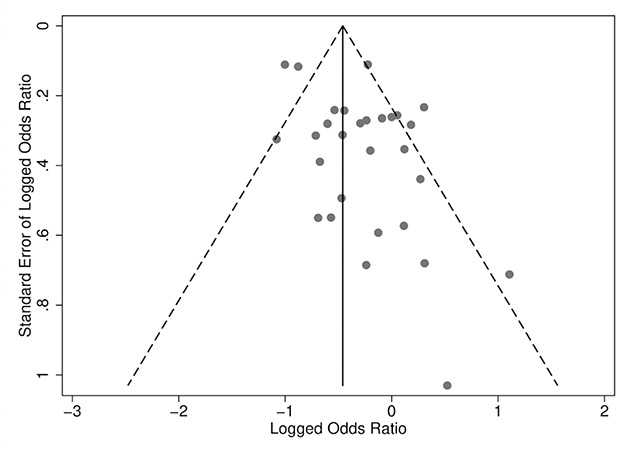
Funnel plot

A second approach to assessing publication bias was a visual examination of a funnel plot (Figure 5). This plot illustrates a slight asymmetry in the funnel with an absence of effects in the lower left part of the funnel. Given the location of the null value relative to the funnel, however, these absent effects are smaller (larger positive effects) than the mean. This helps explain why our third approach, the trim‐and‐fill method suggested no missing effects given that it only assessed for missing effects near or greater than one (Note: using Stata's *metatrim*, this necessitated flipping the sign of the logged odds ratios, changing the direction of the effects).

Although publication selection bias cannot be definitely ruled out, we assess a low risk of our findings resulting from publication selection bias given the large percentage of technical reports in this synthesis.

## Discussion

### Summary of main results

The main objective of this review was to synthesize the evidence on the effectiveness of pre‐charge interventions involving police warning or counsel and release, and cautioning schemes in reducing delinquent behavior. Specifically, we aimed to assess the overall effectiveness of police‐initiated diversions strategies, compare the relative effectiveness of different types of police‐initiated diversion, and finally examine whether effectiveness was related to characteristics of the youth.

The overall results suggest that police‐led diversion modestly reduces future delinquent behavior of low‐risk youth relative to traditional processing. The subset of studies judged to be free from any obvious risk of bias produced an overall mean effect size that was statistically significant and favoured the diversion condition. Analyses based on less methodologically sound studies produced more varied results that were not always statistically significant, but that had an overall mean effect that was roughly similar to that of the higher quality studies. The size of this effect, however, is modest. The overall mean result across all studies translates into a 6 percentage point reduction in reoffending from a benchmark rate of 50 percent.

We were able to categorize the diversion methods used across the studies into one of three based models: diversion only (often called counsel and release), diversion with referral to services, and diversion with police‐led restorative justice. Rather unexpectedly, the effect was modestly larger for the diversion only contrasts, although the differences across the diversion types were not statistically significant. Furthermore, the comparison between diversion only and diversion plus referral‐to‐services modestly favored the latter in a sensitivity analysis that only included studies that compared both of these diversion types to a common traditional processing condition. As such, the existing evidence provides no basis for concluding that there are meaningful differences across the diversion types.

Finally, there was insufficient information and variability across the studies in terms of youth characteristics to allow for examination of differential effects for different types of youth. Thus we were unable to address this aim of the review.

### Overall completeness and applicability of evidence

The positive findings of this review with regard to the benefits of diversion may have limited applicability to current juvenile justice processing. The majority of the evidence is from data collected during the 1970s and 1980s. Analyses showed no clear evidence of differential effectiveness over time, but the number of studies post‐2000 (five studies) is too limited to be certain that the positive results found in this review will continue to hold given shifts in traditional‐processing of that time period. Similarly, most of the evidence is based on data collected in the United States, with a handful of studies from Australia, the UK, and Canada. Thus, these results are clearly only applicable to these countries and additional evidence from outside of the United States is clearly needed to better inform policy in those contexts.

### Quality of the evidence

Overall, the quality of evidence for this review is strong, with a majority of studies using random assignment designs. The availability of official measures of delinquency (e.g., police or court records) reduced problems of attrition and the potential for knowledge of the condition on the part of the youth from affecting outcome measurement. There was no evidence across these studies of selective reporting of outcomes and all studies reported a general measure of delinquency based on official data. Furthermore, the subset of studies judged by us to be at low‐risk of bias produced a homogeneous overall effect favoring the diversion condition.

### Limitations and potential biases in the review process

In general, the description of the delivery of the interventions was poor. In most cases, the reports did not give a sufficient description of the cautioning process that would facilitate a direct replication of the intervention. Specifically, it was rarely clear what was said to diverted juveniles by way of a caution and there was little information that described how the diversion disposal was perceived by the juvenile. Theoretically, a diversion presented as a ‘last chance’ could be perceived very differently from a diversion presented as a ‘helping hand’ or a ‘way out’. The inclusion of this information could have benefitted our understanding of study heterogeneity.

### Agreements and disagreements with other studies or reviews

The review that most closely matches the aims of our review was Wilson and Hoge (2013) who included both pre‐charge and post‐charge diversions, but separated these in their analyses. They found a slightly larger beneficial effect for pre‐charge compared to post‐charge diversions (which we did not test). Wilson and Hoge (2013) did not identify the 11 studies they defined as pre‐charge interventions, but our analysis featured nine of the same references, suggesting that both reviews used rigorous search methods. However, it appears that Wilson and Hoge (2013) treated Lincoln, Teilmann, Klein, and Labin (1977) and Klein (1986) as discrete studies although they reported on the same evaluations: this may have led to a small bias in their results. We screened all references in the Wilson and Hoge (2013) paper for eligibility as part of our review. Consequently, as we included fourteen publications, we are confident that our review is an advancement on their work. Our results are also consistent with the broader Campbell systematic review and meta‐analysis on any form of diversion by Petrosino et al. (2010). Overall, the findings of these three reviews are consistent in their support for the preventive effects of pre‐charge diversion.

## Authors' conclusions

### Implications for practice and policy

The findings from this systematic review support the use of police‐led diversion for low‐risk youth with limited or no prior involvement with the juvenile justice system. The pattern of evidence clearly favors diversion over traditional processing with no indication that diversion programs are harmful, that is, leads to increased delinquency. Although there are caveats to these conclusions that justify some equivocation in our conclusion (e.g., the age of the evidence, variations in statistical significance across different analyses), the pattern of evidence suggests that police departments and policy‐makers should consider diversionary programs as part of the mix of solutions for addressing youth crime.

### Implications for research

The main research need that emerges from this review is contemporary random‐assignment studies comparing police‐led diversion with traditional processing. There are simply too few studies conducted in the last decade to be confident that the positive results will hold for the current context of juvenile justice. Similarly, most of the evidence comes from the United States, indicating a clear need for additional high‐quality research from other countries.

Although we did not systematically catalogue non‐delinquency outcomes across these studies, they were few. This is unfortunate. The potential benefits of police‐led diversion extend beyond reduced‐risk of future delinquent behavior and include such possible benefits as improved attitudes toward the police (e.g., perceptions of police legitimacy), and a greater sense of procedural justice and fairness. Additionally, the lack of penetration into the juvenile justice system may help prevent negative school outcomes. We also were unable to capture race and gender effects, as they were largely unreported in the studies. The need for contemporary random‐assignment studies also includes greater sensitivity to reporting subgroup data by race and gender and other equity criteria, in order for future meta‐analyses to explore these factors as meaningful moderators (see Neyroud & Slothower, 2013).

In the included studies, labelling theory was the dominant theoretical rationale for why diversion might work. This helps explain why much of the research featured in our review is from the 1970s and 1980s; the heyday of labeling theory. Although rarely alluded to in the studies, more contemporary criminological theories also have relevance to diversionary practices: restorative justice and procedural justice in particular. Discretionary police use of diversion could be seen by a youthful offender as an act of ‘fairness’ that supports the perceived legitimacy of police. Alternatively, the demonstration of a restorative‐led diversion may model effective conflict resolution for the youth. Exposure to procedural or restorative justice applied appropriately may form part of the mechanism that explains the reduction in reoffending observed in our review. Future theoretical work should consider applying these theoretical frameworks to diversionary practices to allow for a new wave of empirical research into potential change mechanisms related to diversion.

The role and value of services following diversion remains unclear. Our results found no statistically significant difference in the effectiveness of diversions with and without a referral to services. The diversity of service activities, the limited descriptions of these activities, and insufficient information on the extent of client compliance prevented a detailed analysis of how these factors might have influenced recidivism. Importantly, this prevented distinguishing the effect of diversion from the effect of services. As is common in many evaluation studies that attempt to reflect the realities of juvenile justice, it is the effectiveness of the *referral* to services that is tested, not the effectiveness of the services themselves. While this is the more valuable approach for decision‐making in juvenile justice, it obscures the varied effectiveness of service activities.

The findings of this review are broadly supportive of police‐led diversion's role in reducing juvenile reoffending. To date, no review about the effectiveness of police‐led diversion in reducing adult reoffending exists. A preliminary scoping search by one of the authors has found far fewer evaluations of police‐led diversion schemes for adults than for juvenile offenders. While the factors that influence offending vary between juveniles and adults, they are not entirely independent, suggesting that police‐led diversion may have benefits for adults and should be explored further.

## Information about this review

### Review authors

**Lead review author**

**Table:** 

**Name: David B. Wilson**	** **
Title:	Chair & Professor
Affiliation:	George Mason University
Address:	4400 University Drive, MS4F4
City, State, Province or County:	Fairfax, Virginia
Post code:	22030
Country:	USA
Phone:	(703) 993‐4701
Email:	dwilsonb@gmu.edu
**Co‐author(s)**	** **
**Name: Iain Brennan**	** **
Title:	Reader
Affiliation:	University of Hull
Address:	Wilberforce Building, University of Hull, Cottingham Rd.
City, State, Province or County:	Hull
Post code:	HU6 7RX
Country:	UK
Phone:	+44 (0) 1482 465717
Email:	I.Brennan@hull.ac.uk
**Co‐author(s)**	** **
**Name: Ajima Olaghere**	** **
Title:	Assistant Professor
Affiliation:	Temple University
Address:	1115 Polett Walk, Gladfelter Hall, 5^th^ Floor
City, State, Province or County:	Philadelphia, Pennsylvania
Post code:	19122
Country:	U.S.A.
Phone:	(215) 204‐8271
Email:	aolaghere@temple.edu

### Roles and responsibilities

- Content: David Wilson has extensive background knowledge on juvenile justice programs. Iain Brennan has experience with executing a research grant on a restorative justice program and has recently completed an evaluation of a police‐led diversion scheme. Ajima Olaghere, along with David Wilson, are currently working on a meta‐analysis focusing on trauma‐informed interventions for at‐risk and justice‐involved youth.
- Systematic review methods: David Wilson has extensive expertise in systematic review methods. Ajima Olaghere has worked on meta‐analyses with David Wilson and previously worked on systematic reviews. Iain Brennan has led a systematic review of interventions to reduce violence in licensed premises.
- Statistical analysis: David Wilson has developed tools that are in wide use for performing the statistical analyses related to meta‐analysis. He also authored a book on these methods with Mark Lipsey.
- Information retrieval: David Wilson, Ian Brennan, and Ajima Olaghere all have experience performing systematic searches on various topics and retrieving studies and documents for review.

### Sources of support

This systematic review is supported by funding from the Jacobs Foundation.

### Declarations of interest

We have no potential conflicts of interest with respect to this review.

### Plans for updating the review

This review will be updated every four years and updating it will be the primary responsibility of David Wilson unless all authors agrees that another author take primary responsibility.

### Data and analyses

Supplemental materials for this study can be found on the Campbell Collaboration website. These materials concern a compressed folder of data files (ASCII files), scripts, and syntax developed for all data compilation and analyses conducted in this review.

## Study Level Coding Form

This coding form is for each unique study. Note that a study may be reported in multiple manuscripts (publications, technical reports, etc.). Also, some reports may include the results for distinct studies, such as evaluations in different cities. Our unit‐of‐analysis for the meta‐analysis is an independent study. No two studies should include any of the same participants. If there are multiple publications for the same study, use the most complete study as the primary study ID and all other related studies as cross reference IDs.

**Table:**

**Identifiers**			
1.	Reference ID	studyid	|__|__|__|__|
2.	Other related references	crossref1	|__|__|__|__|
		corssref2	|__|__|__|__|
		corssref3	|__|__|__|__|
		corssref4	|__|__|__|__|
		corssref5	|__|__|__|__|
3.	Coder's initials	sinitials	|__|__|__|
4.	Creation date (mm/dd/yy)	sdate	|__|__|__|__|__|
5.	Modification date (mm/dd/yy)	sdatem	|__|__|__|__|__|
**General Study Information**			
6.	Publication type 1. Book 2. Journal article/book chapter 3. Thesis‐dissertation 4. Technical report 5. Conference paper 6. Other	pubtype	|__|
7.	Geographic location of study 1. United States 2. Canada 3. UK 4. Australia 5. EU 6. Other	location	|__|
8.	Years of data collection		
	Year data collection started	datastart	|__|__|__|__|
	Year data collection ended	dataend	|__|__|__|__|
9.	Researcher involvement 1. CJ system initiated diversion; internal evaluator 2. CJ system initiated diversion; external evaluator 3. Researcher initiated diversion program	resinvolve	|__|
10.	Was this research funded by a grant or external agency (0=no; 1=yes; 9=cannot tell)	funding	|__|
**Research Design**			
11.	Unit of assignment to conditions 1. individual 2. incident (might include multiple youth) 3. officer 4. police station or jurisdiction 5. other 9. cannot tell	uoa	|__|
12.	How subjects were assigned to condition (this is about assignment not sampling) 1. randomly after matching, yoking, stratification, blocking, etc. 2. randomly without matching 3. regression discontinuity (quantitative cutting point defines groups) 4. wait list control or other such quasi‐random procedures (e.g., alternating cases) 5. quasi‐experimental, matched individual level 6. quasi‐experimental, matched group level (e.g., classrooms) 7. quasi‐experimental, statistical controls for baseline differences 8. quasi‐experimental, no statistical controls for baseline differences 9. quasi‐experimental, other 10. quasi‐experimental, cohort design (historical controls)	design	|__|
13.	If random assignment or regression discontinuity design: 1. integrity of randomization or other assignment method maintained (no more than a few cases failed to end up in desired group) 2. failures of randomization or assignment occurred 3. no information on integrity of assignment process	rndinteg	|__|
14.	[RISK OF BIAS ITEM] Is there any risk of selective outcome reporting bias, that is, is there any evidence that the authors have not reported findings for all variables measured as part of this study? (1=low risk; 2=high risk; 3=unclear risk)	selectreport	|__|
15.	Study level coding notes	snotes	

## Comparison Level Coding Form

This coding form is for each treatment/comparison contrast coded from a study. For most studies, you will only code this form once. However, some studies may have two or more treatment conditions or two or more comparison conditions. In the coding below, it is critical to indicate if any of the treatment/comparison contrasts for a study share sample participants. For example, a study might have two distinct treatments but only one comparison group. In this case, these comparisons share sample participants (i.e., the same comparison condition).

**Table:**

**Identifiers**		
1. Reference ID	studyid	|__|__|__|__|
2. Condition ID	compid	|__|__|__|__|
3. Coder's initials	cinitials	|__|__|__|
4. Creation date (mm/dd/yy)	cdate	|__|__|__|__|__|
5. Modification date (mm/dd/yy)	Cdatem	|__|__|__|__|__|
6. Treatment group label	txlabel	|__|__|__|__|__|
7. Control/comparison group label	cglabel	|__|__|__|__|__|
		
**Sample Information**		
8. Treatment group sample size (at start of study before attrition; ‐99999 if cannot tell)	ctxn	|__|__|__|__|__|
9. Comparison group sample size (at start of study before attrition; ‐99999 if cannot tell)	ccgn	|__|__|__|__|__|
10. Mean or median age of sample (99.9 if cannot tell)	meanage	|__|__|.__|
11. Youngest age in sample (‐99 if cannot tell)	minage	|__|__|
12. Oldest age in sample (‐99 if cannot tell)	maxage	|__|__|
13. Sex distribution for this treatment/comparison contrast 1. 100% Male 2. 90‐99% Male 3. 75‐89% Male 4. 26‐75% Male 5. 11‐25% Male 6. 1‐10% Male 7. 0% Male 9. Unknown	sex	|__|
14. Percent of this condition that is represented by each of the following race/ethnic group (‐99.9 if missing unknown):		
White	white	|__|__|__|.__|
African American	black	|__|__|__|.__|
Hispanic (non‐White)	hispanic	|__|__|__|.__|
Asian	asian	|__|__|__|.__|
Other	raceother	|__|__|__|.__|
**Nature of Treatment Condition**		
15. Type of diversion (1=traditional cautioning; 2=caution plus, 3=police restorative cautioning, 4=final warning or reprimand, 5=other) [Note: we will add to the list of options as we code studies.]	diversion	|__|
16. Referral to services (0=no; 1=yes (external agencies); 2=yes (internal to CJ); 9=cannot tell)	referral	|__|
17. Other elements of this condition:	txother	
**Nature of Comparison Condition**		
18. Type of comparison condition (1=formal court processing not otherwise specified; 2=probation; 3=adjudicated youth; 4=other) [Note: we will add to the list of options as we code studies.]	comparison	|__|
19. Services or sanctions for the comparison condition	cgother	
**Comparability of Conditions**		
20. Were the conditions compared for baseline equivalence on any of the following, either statistically or descriptively? (0=no; 1=yes; 9=cannot tell)		
sex	basediff1	|__|
race	basediff2	|__|
age	basediff3	|__|
delinquency history and/or delinquency risk	basediff4	|__|
21. RISK OF BIAS ITEM: Based on the above, is there a risk of selection bias, that is, that the groups were different at baseline? (1=low risk; 2=high risk; 3=unclear)	selectbias	|__|
22. RISK OF BIAS ITEM: Is there a risk of general attrition bias for the primary outcome measure, that is, attrition in excess of 10%? (1=low risk; 2=high risk; 3=unclear)	attrition1	|__|
23. RISK OF BIAS ITEM: Is there a risk of different attrition bias for the primary outcome measure, that is, meaningful differential attrition? (1=low risk; 2=high risk; 3=unclear)	attrition2	|__|
**Notes**		
Notes about coding this comparison	cnotes	

## Outcome (Dependent Variable) Coding Form

Code each eligible outcome or dependent variable using the form below. Note that you should code this only once for a variable that is measured at multiple time points. That is, recidivism measured at 3, 6, and 9‐months is a single dependent variable. Code the characteristics of the measure using this form and the data for each measurement time point on the effect size forms.

**Table:**

**Identifiers**		
1. Reference ID	studyid	|__|__|__|__|
2. Coder's initials	dvinitials	|__|__|__|
3. Creation date (mm/dd/yy)	dvdate	|__|__|__|__|__
4. Modification date (mm/dd/yy)	dvdatem	|__|__|__|__|__
5. Outcome ID	dvid	|__|__|__|__|
6. Dependent variable label	dvlabel	|__|__|__|__|
		
**Characteristics of Variable**		
7. Elements reported in this delinquency measure irrespective of the type of incident and reporting source (check best one):	dvelements	|__|__|__|
1. global dichotomy or polychotomy (e. g., offended or recidivated, yes/no), most common for arrests/convictions		
2. summed dichotomous (e.g., sum of yes/no on list of specific offenses), almost never see, composite of dichotomy or polychotomy elements		
3. frequency or rate, (count of incident; incidents per 1000 persons)		
4. severity (seriousness rating or index), see this often with self‐report measures		
5. event timing (e.g., days without recidivism; time to first offense)		
6. proportion or amount of time in custody, under supervision, etc., not seen often		
7. rating of amount of delinquency, severity, change, etc.(this is similar to frequency but in rating form, ex. how often you did“x” behavior)		
8. more than one of above elements combined in composite measure		
9. other		
99. cannot tell		
8. Type of delinquency/recidivism represented by this measure (what's counted, irrespective of source of information and authors' label or description of the measure) check best one:	dvtype	|__|__|__|
1. antisocial behavior, not specifically restricted to criminally delinquent acts		
2. unofficial delinquent behavior, e.g., from self or observer's report		
3. school disciplinary actions relating to delinquent/antisocial behavior		
4. arrests or police contacts		
5. probation contact, violations, actions, etc.		
6. court contact, actions, petitions, convictions, appearances		
7. parole contact, violations, action, etc., excluding re‐institutionalization		
8. institutional disciplinary actions or institutional behavior		
9. institutionalization or re‐institutionalization		
10. other		
99. cannot tell		
9. Definitional boundary for measure (select best options)	dvcrime	|__|__|__|
1. all “offenses” included		
2. substance abuse only		
3. property crime only		
4. person crimes only (victim personally involved in crime)		
5. status offenses only		
6. criminal offenses only, i.e., all but status offenses		
10. other		
99. cannot tell		
10. Source of delinquency measure	dvsource	|__|__|__|
1. self‐report: paper & pencil or computer		
2. self‐report: personal interview		
3. self‐report: telephone interview		
4. self‐report: other		
5. self‐report: cannot tell		
6. other report: parent		
7. other report: peers		
8. other report: teacher(s)		
9. other report: therapist/service provider		
10. other report: other		
11. other report: cannot tell		
12. records: school		
13. records: police		
14. records: probation		
15. records: court		
16. records: custodial institution		
17. records: regional crime statistics		
18. records: other		
19. records: cannot tell		
20. any other		
99. cannot tell		
11. RISK OF BIAS ITEM: Person providing outcome data knows which condition the participant is in (i.e., is there a potential bias from the lack of blinding of the assessor?) (1=low risk; 2=high risk; 3=unclear risk)	dvbias	|__|
12. Notes regarding this outcome measure	dvnotes	

## Effect Size Coding Form

Code all effect sizes of interest using the form below, coding each effect size separately (i.e., with a different copy of the form or record in the database). Indicate the study ID, the comparison ID, and the dependent variable ID. Give each effect size within a study a unique idea, numbering sequentially (1, 2, 3 …).

There are several ways to compute effect sizes using the different tabs. ONLY USE ONE METHOD per effect size. If you have the raw means and also a regression coefficient for the same outcome from a model that adjusts for baseline differences, these are two different effect sizes. The different effect size computation methods are:

1. Means and standard deviations
2. Means and standard errors
3. Frequency of failures in each condition
4. Proportion of failures in each condition
5. Logistic regression coefficient for treatment effect dummy code
6. OLS unstandardized regression coefficient
7. OLS standardized regression coefficient
8. Independent samples t‐test
9. Chi‐square test (2 by 2, df = 1)
10. Point‐biserial correlation coefficient
11. Phi correlation coefficient
12. Hand computation (e.g., using the online effect size calculator)

**Table:**

**Identifiers**		
1. Reference ID	studyid	|__|__|__|__|
2. Coder initials	esinitials	|__|__|__|
3. Creation date	esdate	|__|__|__|__|__
4. Modification date	esdatem	|__|__|__|__|__
5. Comparison ID	compid	|__|__|__|__|
6. Outcome ID	dvid	|__|__|__|__|
7. Effect Size ID	esid	|__|__|__|__|
**Effect Size Information**		
8. Direction of effect (1=favours treatment; 2=favours control; 3=neither, exactly equal; 9=cannot tell)	esdirect	|__|
9. Type of effect size (i.e., baseline differences, first post treatment outcome measure, or a follow‐up measure) (1=baseline (pre‐test); 2=post‐test; 3=follow‐up)	estype	|__|
10. Effect reported as statistically significant by authors (1=yes; 0=no; 9=cannot tell)	essig	|__|
11. Timing of measurement (months captured by the measure from the point of assignment to conditions or diversion/formal processing; if reported in months, divide by 4.3; 8888 if not applicable; 9999 if missing)		
Mean	estime1	|__|__|__|__|
Minimum	estime2	|__|__|__|__|
Maximum	estime3	|__|__|__|__|
**Effect Size Data**		
12. Treatment group sample size for this effect size	estxn	|__|__|__|__|
13. Comparison group sample size for this effect size	escgn	|__|__|__|__|
***Scaled outcome data***		
14. Mean treatment group	esmtx	|__|__|__|__|.__|__|
15. Mean comparison group	esmcg	|__|__|__|__|.__|__|
16. Are the above means adjusted for baseline differences? (1=yes; 0=no; 9=cannot tell)	esmadj	|__|
17. Standard deviation treatment group	essdtx	|__|__|__|__|.__|__|
18. Standard deviation comparison group	essdcg	|__|__|__|__|.__|__|
19. Standard error treatment group	essetx	|__|__|__|__|.__|__|
20. Standard error comparison group	essecg	|__|__|__|__|.__|__|
***Dichotomous outcome data***		
21. Treatment group number successful	Estxn	|__|__|__|__|
22. Comparison group number successful	Escgn	|__|__|__|__|
23. Treatment group number failures	estxnf	|__|__|__|__|
24. Comparison group number failures	escgnf	|__|__|__|__|
25. Treatment group proportion of successes (only code this if raw frequencies are not available)	estxpf	|__|.__|__|__|__|__|
26. Comparison group proportion of successes (only code this if raw frequencies are not available)	escgpf	|__|.__|__|__|__|__|
27. Are the above frequencies or proportions adjusted for baseline differences? (1=yes; 0=no; 9=cannot tell)	espadj	|__|
***Logistic regression***		
28. Logistic regression coefficient (for treatment effect dummy)	eslgor	|__|.__|__|__|__|__|
29. Standard error for logistic regression coefficient	esselgor	|__|.__|__|__|__|__|
30. *t*‐test or *z*‐test for logistic regression coefficient	esolst	|__|.__|__|__|__|__|
31. Odds ratio for treatment effect dummy (optional)	esor	|__|__|__|.__|__|__|
***OLS regression***		
32. Unstandardized regression coefficient	esolsb	|__|.__|__|__|__|__
33. Standard regression coefficient	esolsbeta	|__|.__|__|__|__|__
34. Standard error of regression coefficient	esolsse	|__|.__|__|__|__|__
35. Standard deviation for dependent variable	essd	|__|.__|__|__|__|__
***Other possible effect size data***		
36. *t*‐test (comparing two‐sample means; not the *t* from a regression model)	est	|__|__|__|__|.__|__|
37. *p‐*value from a *t*‐test (comparing two‐sample means; not the *t* from a regression model)	espfromt	|__|.__|__|__|__|__|
38. Correlation coefficient point‐biserial (treatment versus comparison correlated with scaled variable)	esrpb	|__|.__|__|__|__|__|
39. Correlation coefficient phi (treatment versus comparison correlated with a dichotomous variable)	esrphi	|__|.__|__|__|__|__|
40. Chi‐square (treatment versus comparison correlated with a dichotomous variable, *df* must equal 1)	eschisq	|__|__|__|__|.__|__|
***Effect size computed by hand (e.g., using online calculator)***		
41. Standardized mean difference effect size computed by hand (*d*‐type)	eshand	|__|__|.__|__|__|__|
42. Variance for standardized mean different effect size computed by hand	eshandv	|__|__|.__|__|__|__|
43. Computed effect size	escalc	|__|__|.__|__|__|__|
44. Computed effect size standard error	escalcse	|__|__|.__|__|__|__|
***Effect size coding notes***		
45. Page number where effect size data found	espage	|__|__|__|__|__|__|
46. Notes about this effect size	esnotes	

## Appendix

**Table:**

**Database:**	Criminal Justice Database
	
**Date:**	September 15, 2016
	
**Search terms:**	juvenile AND (caution* OR “caution plus” OR “restorative caution” OR “final warning” OR reprimand) AND evaluat* AND recidivism
	
**Yield:**	1,278
	
**Notes**	Searched using the command line
	
**Database:**	Criminal Justice Abstracts
** **	
**Date:**	September 9, 2016 ‐ September 10, 2016 Time: 10:22 AM EST
** **	
**Search terms:**	(youth OR child OR juvenile OR delinquent OR devian* OR student OR adolescent OR “young person” OR “young offender*” OR bully* OR “youthful offender”) **OR** (diverted OR diversion OR caution* OR “caution plus” OR restorative OR “restorative caution” OR triage OR “final warning” OR reprimand OR “alternative* to custody” OR “pre‐charge” OR “pre‐caution” OR “pre‐court” OR “pre‐custody” OR “alternative program*” OR disposal OR disposition OR liaison OR Police‐led OR police OR “law enforcement”) **OR** (outcome OR evaluat* OR effect OR effectiv* OR experiment* OR quasi OR assessment OR RCT OR “random* control*”) **OR** (recidivism OR arrest* OR rearrest* OR citation OR offend* OR reoffend* OR conviction OR reconviction OR adjudication OR adjudicate)
**Yield:**	1,539
** **	
**Notes:**	9/9/2016: Locked out the database for 1 hour for “exceeded the maximum downloads per session threshold.” Stopped at 16 (need to download) at 50 references per page.
	9/10/2016: resumed search; locked out for 1 hour for same reason as above. Stopped at page 30 (need to download) at 50 references per page.
**Database (source):**	Peter Neyroud
**Date:**	6/22/2016 (date received)
** **	
**Search terms:**	n/a; references forwarded by Mr. Neyroud
** **	
**Yield:**	13
** **	
**Notes:**	Provided by Mr. Neyroud; references retrieved from Google Scholar
	
**Database:**	ProQuest Dissertations & Theses Global
**Date:**	September 19, 2016
** **	
**Search terms:**	(delinquent) AND (“law enforcement” OR diversion) AND (evaluat*) AND (recidivism)
** **	
**Yield:**	4,884
	
**Database:**	Sociological Abstracts
** **	
**Date:**	October 20, 2016
** **	** **
**Search terms:**	(youth OR child OR juvenile OR delinquent OR devian* OR student OR adolescent OR “young person” OR “young offender*” OR bully* OR “youthful offender”) AND (diverted OR diversion OR caution* OR “caution plus” OR restorative OR “restorative caution” OR triage OR “final warning” OR reprimand OR “alternative* to custody” OR “pre‐charge” OR “pre‐caution” OR “pre‐court” OR “pre‐custody” OR “alternative program*” OR disposal OR disposition OR liaison OR Police‐led OR police OR “law enforcement” OR “civil citation”) AND (outcome OR evaluat* OR effect OR effectiv* OR experiment* OR quasi OR assessment OR RCT OR “random* control*”) AND (recidivism OR arrest* OR rearrest* OR citation OR offend* OR reoffend* OR conviction OR reconviction OR adjudication OR adjudicate)
** **	
**Yield:**	1,099
	
**Database:**	HeinOnline (Law Journal Library)
** **	** **
** **	**Search 1**
** **	
**Date:**	Jan 5, 2017
** **	
**Search terms:**	(diversion OR caution) AND (police OR “law enforcement”) AND (effectiveness OR experiment OR outcome) AND (young OR juvenile OR delinquent) AND (redicivism OR arrest* OR rearrest* OR reoffend*)
** **	
**Yield:**	1,209
** **	
**Notes:**	Limited to ‘Articles’ and ‘Criminal Justice’ Re‐ran search after being locked out from
** **	
**Database:**	HeinOnline Criminal Justice Journals
** **	
** **	**Search 2**
** **	
**Search terms:**	(diversion OR caution) AND (police OR “law enforcement”) AND (effectiveness OR experiment OR outcome) AND (young OR juvenile OR delinquent) AND (recidivism OR arrest* OR rearrest* OR reoffend*)
** **	
**Yield:**	2,212
** **	
**Date:**	Jan 5, 2017
** **	
**Notes:**	Main HeinOnline Search; Enter search terms; Articles; Criminal Justice Journals 18:15 5/1/17 1‐1399 (n=1085)
	
**Database:**	GoogleScholar
** **	
**Date:**	8/4/2016
** **	
**Search terms:**	police diversion
** **	
**Yield:**	114,000
** **	** **
**Notes:**	Will retrieve the first 500 references
	
**Database:**	NCJRS
** **	
**Date:**	Jan 13, 2017
** **	
**Search terms:**	diversion juvenile “law enforcement” [All terms; Publications (Full text)]
** **	
**Yield:**	171
	
**Database:**	OVID
** **	** **
**Date:**	October 20, 2016
** **	
**Search terms:**	(youth or child or delinquent or juvenile) and (diversion or police or “law enforcement”) and (outcome or evaluat*) and (recidivism or rearrest)
** **	
**Yield:**	833
** **	
**Notes:**	Selected “Journals @OVID Full Text”
	
** **	**Database:** PolicyArchive
** **	
** **	**Date:** October 20, 2016
** **	** **
** **	**Search terms:** juvenile
** **	
** **	**Yield:** 120
** **	
** **	**Database:** PolicyFile
** **	
** **	**Date:** October 20, 2016
** **	
** **	**Search terms:** juvenile OR delinquent AND outcome OR eval* AND recidivism
** **	
** **	**Yield:** 360
	
** **	**Database:** SSRN
** **	
** **	**Date:** Jan 17, 2017
** **	
** **	**Search terms:** diversion police
** **	
** **	**Yield:** 17
	
** **	**Database:** PsycInfo
** **	
** **	**Date:** January 5, 2017
** **	
** **	**Search terms:** (youth OR child OR juvenile OR delinquent OR devian* OR student OR adolescent OR “young person” OR “young offender*” OR bully* OR “youthful offender”) AND (diverted OR diversion OR caution* OR “caution plus” OR restorative OR “restorative caution” OR triage OR “final warning” OR reprimand OR “alternative* to custody” OR “pre‐charge” OR “pre‐caution” OR “pre‐court” OR “pre‐custody” OR “alternative program*” OR disposal OR disposition) AND (outcome OR evaluat* OR effect OR effectiveness OR experiment* OR quasi OR assessment OR RCT OR “random* control*”) AND (recidivism OR arrest* OR rearrest* OR offend* OR reoffend* OR conviction OR reconviction OR adjudication) AND (police OR “law enforcement”)
** **	
** **	**Yield:** 81
	
**Database:**	CINCH (via Informit). Not specifically Australian Institute database
** **	
**Date:**	January 10, 2017
** **	
**Search terms:**	**#5** (ALLTERMS,FC:diversion OR ALLTERMS,FC:caution) AND (ALLTERMS,FC:police OR ALLTERMS,FC:”law enforcement”) AND (ALLTERMS,FC:effectiveness OR ALLTERMS,FC:experiment OR ALLTERMS,FC:outcome) AND (ALLTERMS,FC:young OR ALLTERMS,FC:juvenile OR ALLTERMS,FC:delinquent) AND (ALLTERMS,FC:recidivism OR ALLTERMS,FC:arrest* OR ALLTERMS,FC:rearrest* OR ALLTERMS,FC:reoffend*) **515** **#4** (diversion OR caution) AND (police OR “law enforcement”) AND (effectiveness OR experiment OR outcome) AND (young OR juvenile OR delinquent) AND (recidivism OR arrest* OR rearrest* OR reoffend*) **0** **#3** ((diversion OR caution)) AND ((police OR “law enforcement”)) AND ((effectiveness OR experiment OR outcome)) AND ((young OR juvenile OR delinquent)) AND ((recidivism OR arrest* OR rearrest* OR reoffend*)) **0** **#2** (ALLTERMS,FC:youth OR ALLTERMS,FC:child OR ALLTERMS,FC:juvenile OR ALLTERMS,FC:delinquent OR ALLTERMS,FC:devian* OR ALLTERMS,FC:student OR ALLTERMS,FC:adolescent OR ALLTERMS,FC:“young OR ALLTERMS,FC:person” OR ALLTERMS,FC:“young OR ALLTERMS,FC:offender*” OR ALLTERMS,FC:bully* OR ALLTERMS,FC:“youthful OR ALLTERMS,FC:offender”) AND (ALLTERMS,FC:diverted OR ALLTERMS,FC:diversion OR ALLTERMS,FC:caution* OR ALLTERMS,FC:“caution OR ALLTERMS,FC:plus” OR ALLTERMS,FC:restorative OR ALLTERMS,FC:“restorative OR ALLTERMS,FC:caution” OR ALLTERMS,FC:triage OR ALLTERMS,FC:“final OR ALLTERMS,FC:warning” OR ALLTERMS,FC:reprimand OR ALLTERMS,FC:“alternative* OR ALLTERMS,FC:to OR ALLTERMS,FC:custody” OR ALLTERMS,FC:“pre‐charge” OR ALLTERMS,FC:“pre‐caution” OR ALLTERMS,FC:“pre‐court” OR ALLTERMS,FC:“pre‐custody” OR ALLTERMS,FC:“alternative OR ALLTERMS,FC:program*” OR ALLTERMS,FC:disposal OR ALLTERMS,FC:disposition OR ALLTERMS,FC:liaison) AND (ALLTERMS,FC:police OR ALLTERMS,FC:”law enforcement”) AND (ALLTERMS,FC:outcome OR ALLTERMS,FC:evaluat* OR ALLTERMS,FC:effect OR ALLTERMS,FC:effectiv*eness OR ALLTERMS,FC:experiment* OR ALLTERMS,FC:quasi OR ALLTERMS,FC:assessment OR ALLTERMS,FC:RCT OR ALLTERMS,FC:“random* OR ALLTERMS,FC:control*”) AND (ALLTERMS,FC:recidivism OR ALLTERMS,FC:arrest* OR ALLTERMS,FC:rearrest* OR ALLTERMS,FC:citation OR ALLTERMS,FC:offend* OR ALLTERMS,FC:reoffend* OR ALLTERMS,FC:conviction OR ALLTERMS,FC:reconviction OR ALLTERMS,FC:adjudication OR ALLTERMS,FC:adjudicated) **7318** **#1** diversion **477**
**Yield:**	515
**Notes:**	Couldn't extract results directly into Zotero, so saved as text files to import into EndNote and transfer to Zotero.
	
**Database:**	First Search
** **	** **
**Date:**	September 26, 2016
** **	
**Search terms:**	(youth OR child OR juvenile OR delinquent OR deviant OR student OR adolescent) AND (diverted OR diversion OR caution OR restorative OR triage OR reprimand OR disposal OR disposition OR liaison OR Police‐led OR police) AND (outcome OR evaluate OR effect OR effective OR experiment
** **	OR quasi OR assessment OR RCT) AND (recidivism OR arrest OR rearrest OR citation OR offend OR reoffend OR conviction OR reconviction OR adjudication OR adjudicate)
** **	
**Yield:**	715
** **	
**Notes:**	Downloaded yield from each database separately
	
**Database:**	Social Services Abstracts
** **	
**Date:**	October 20, 2016
** **	
**Search terms:**	(youth OR child OR juvenile OR delinquent OR devian* OR student OR adolescent OR “young person” OR “young offender*” OR bully* OR “youthful offender”) AND (diverted OR diversion OR caution* OR “caution plus” OR restorative OR “restorative caution” OR triage OR “final warning” OR reprimand OR “alternative* to custody” OR “pre‐charge” OR “pre‐caution” OR “pre‐court” OR “pre‐custody” OR “alternative program*” OR disposal OR disposition OR liaison OR Police‐led OR police OR “law enforcement” OR “civil citation”) AND (outcome OR evaluat* OR effect OR effectiv* OR experiment* OR quasi OR assessment OR RCT OR “random* control*”) AND (recidivism OR arrest* OR rearrest* OR citation OR offend* OR reoffend* OR conviction OR reconviction OR adjudication OR adjudicate)
**Yield:**	258
	
**Database:**	Worldwide Political Science Abstracts
** **	
**Date:**	October 20, 2016
** **	
**Search terms:**	(youth OR child OR juvenile OR delinquent OR devian* OR student OR adolescent OR “young person” OR “young offender*” OR bully* OR “youthful offender”) AND (diverted OR diversion OR caution* OR “caution plus” OR restorative OR “restorative caution” OR triage OR “final warning” OR reprimand OR “alternative* to custody” OR “pre‐charge” OR “pre‐caution” OR “pre‐court” OR “pre‐custody” OR “alternative program*” OR disposal OR disposition OR liaison OR Police‐led OR police OR “law enforcement” OR “civil citation”) AND (outcome OR evaluat* OR effect OR effectiv* OR experiment* OR quasi OR assessment OR RCT OR “random* control*”) AND (recidivism OR arrest* OR rearrest* OR citation OR offend* OR reoffend* OR conviction OR reconviction OR adjudication OR adjudicate)
** **	
**Yield:**	59
	
**Website:**	Home Office (https://www.gov.uk/government/organisations/home‐office/about/research) (http://webarchive.nationalarchives.gov.uk/20110218135832/http:/rds.homeoffice.gov.uk/rds/pubsintro1.html)
** **	
**Date:**	January 15, 2017
** **	
**Search terms:**	(Home Office): youth OR juvenile OR delinquent AND diversion OR caution OR reprimand OR police OR police‐led AND outcome OR experiment
** **	
**Yield:**	134 (Home Office) + 338 (Home Office Archives) = 472 total
** **	
**Notes:**	Searched the section on Home Office research website and the archived RDS website. Searched each site using the search toolbar and filtered by “Home Office.” Searched the Home Office archives by browsing by subject and capturing relevant titles under relevant subject headings via Zotero webpage capture. The search field on the archive website did not work; routed to an error page whenever a search was attempted. Counted the number of key publications listed under each subject as the total yield. This was often hyperlinked documents nested within a description of the subject category or listed under “key publications.” Subject categories selected included: British crime survey: extension to 10 and 15 year olds; British crime survey and other surveys; Burglary; Changing behavior to prevent crime; Organized crime; Crime reduction research series; Drug use and young people; Drugs and offending; Police community relations; Police powers and procedures; Police research series; Policing and reducing crime unit briefing notes; ad hoc policing and reducing crime unit publications; policing methods and approaches; policing powers and procedures; robbery and street crime; anti‐social behavior
	
**Website:**	Ministry of Justice (http://www.justice.gov.uk/)
** **	
**Date:**	January 15, 2017
** **	
**Search terms:**	juvenile AND police
** **	
**Yield:**	23
	
**Database:**	Google Scholar
** **	
**Search terms:**	(youth OR juvenile OR delinquent OR adolescent) AND (diversion OR caution OR restorative OR “alternative to custody” OR disposal) AND (police OR “law enforcement”) AND (outcome OR evaluation OR effective) AND (recidivism OR arrest OR offense OR conviction)
** **	
**Date:**	18 August 2016
** **	
**Yield:**	151,000
	
**Database:**	HeinOnline Criminal Justice Journals
** **	** **
**Date:**	Jan 5, 2017
** **	
**Search terms:**	(diversion OR caution) AND (police OR “law enforcement”) AND (effectiveness OR experiment OR outcome) AND (young OR juvenile OR delinquent) AND (recidivism OR arrest* OR rearrest* OR reoffend*)
** **	
**Yield:**	2,212
**Notes:**	Main HeinOnline Search; Enter search terms; Articles; Criminal Justice Journals 18:15 5/1/17 1‐1,399 (n=1085)
	
**Database:**	International Bibliography of the Social Sciences
** **	
**Search terms:**	diversion AND (police OR “law enforcement”)
** **	
**Yield:**	38
	
**Database:**	ProQuest Dissertations and Theses A&I
** **	
**Date:**	August 16, 2016
** **	
**Search terms:**	(youth OR child OR juvenile OR delinquent OR devian* OR student OR adolescent OR “young person” OR “young offender*” OR bully* OR “youthful offender”) AND (diverted OR diversion OR caution* OR “caution plus” OR restorative OR “restorative caution” OR triage OR “final warning” OR reprimand OR “alternative* to custody” OR “pre‐charge” OR “pre‐caution” OR “pre‐court” OR
** **	“pre‐custody” OR “alternative program*” OR disposal OR disposition) AND (outcome OR evaluat* OR effect OR effectiveness OR experiment* OR quasi OR assessment OR rct OR “random* control*”) AND (recidivism OR arrest* OR rearrest* OR offend* OR reoffend* OR conviction OR recondition OR adjudication) AND (police OR “law enforcement”)
** **	
**Yield:**	518
	
**Database:**	ProQuest Dissertations and Theses UK and Ireland
** **	
**Date:**	August 11, 2016
** **	
**Search terms**:	(youth OR child OR juvenile OR delinquent OR devian* OR student OR adolescent OR “young person” OR “young offender*” OR bully* OR “youthful offender”) AND (diverted OR diversion OR caution* OR “caution plus” OR restorative OR “restorative caution” OR triage OR “final warning” OR reprimand OR “alternative* to custody” OR “pre‐charge” OR “pre‐caution” OR “pre‐court” OR “pre‐custody” OR “alternative program*” OR disposal OR disposition OR liaison) AND (police OR “law enforcement”) AND (outcome OR evaluat* OR effect OR effectiv*eness OR experiment* OR quasi OR assessment OR RCT OR “random* control*”) AND (recidivism OR arrest* OR rearrest* OR citation OR offend* OR reoffend* OR conviction OR reconviction OR adjudication OR adjudicated)
** **	
**Yield:**	438
	
**Database:**	PubMed
** **	
**Date:**	January 17, 2017
** **	
**Search terms:**	“pre‐court” OR “pre‐custody” OR “alternative program*” OR disposal OR disposition OR liaison) AND (police OR “law enforcement”) AND (outcome OR evaluat* OR effect OR effectiv* OR experiment* OR quasi OR assessment OR RCT OR “random* control*”) AND (recidivism OR arrest* OR rearrest* OR citation OR offend* OR reoffend* OR conviction OR reconviction OR adjudication OR adjudicated)
** **	
**Yield:**	26
	
**Database:**	RAND Documents
** **	
**Search terms:**	diversion (Filters: Research; All Topics; All Time)
** **	
**Date:**	August 18, 2016
** **	
**Yield:**	48
	
**Database:**	safetylit.org
** **	** **
**Search terms:**	Diversion AND police OR law enforcement (text word and synonyms)
** **	
**Yield:**	54
	
**Database:**	EconLit
** **	
**Date:**	August 2, 2016
** **	
**Search terms (SmartText):**	youth OR child OR juvenile OR delinquent OR devian* OR student OR adolescent OR “young person” OR “young offender*” OR bully* OR “youthful offender” AND diverted OR diversion OR caution* OR “caution plus” OR restorative OR “restorative caution” OR triage OR “final warning” OR reprimand OR “alternative* to custody” OR “pre‐charge” OR “pre‐caution” OR “pre‐court” OR “pre‐custody” OR “alternative program*” OR disposal OR disposition OR liaison AND police OR “law enforcement” AND outcome OR evaluat* OR effect OR effectiv* OR experiment* OR quasi OR assessment OR RCT OR “random* control*” AND recidivism OR arrest* OR rearrest* OR citation OR offend* OR reoffend* OR conviction OR reconviction OR adjudication OR adjudicated
** **	
**Yield:**	1, 542
	
**Database:**	Australian Institute of Criminology (informit)
** **	
**Search terms:**	diversion OR caution AND police OR “law enforcement” AND effectiveness OR experiment OR outcome AND young OR juvenile OR delinquent AND recidivism OR arrest* OR rearrest* OR reoffend*
** **	
**Yield:**	515
	
**Database:**	Australian Institute of Criminology
** **	
**Date:**	Oct 4, 2016
** **	
**Search terms:**	diversion & police & juvenile & evaluation Category: Publications only
** **	** **
**Yield:**	295
	
**Database:**	Australian Institute of Criminology
** **	** **
**Date:**	13/1/17
** **	
**Search terms:**	diversion juvenile police [Match: All search words; Category: Publications]
** **	
**Yield:**	295
	
**Database:**	Google Scholar
** **	
**Date:**	January5, 2017
** **	
**Search terms:**	(diversion OR caution) AND (police OR “law enforcement”) AND (effectiveness OR experiment OR outcome) AND (young OR juvenile OR delinquent) AND (recidivism OR arrest* OR rearrest* OR reoffend*)
** **	
**Yield:**	65,900
** **	
**Notes:**	16:00 5/1/17: Pages 1‐5 (n=100)
** **	16:34 5/1/17: Pages 5‐17 (n=221)
** **	16:41 5/1/17: Pages 18‐24 (n=291)
** **	16:43 5/1/17: Pages 25‐27 (n=321)
** **	15:22 6/1/17: Pages 28‐34 (n=391)

## Appendix

**Table 1:** Study publication type, location, years data collected, unit of analysis and condition assignment

**Authorship (Year)**	** **	**Publication type**	**Location**	**Years data collected**	**Unit of analysis**	**Condition assignment**
Byles & Maurice (1979)		Journal	Hamilton, Ontario, Canada	1973‐1977	Individual	Random without matching
Cunningham (2007)		Agency report	Northern Territory, Australia	2000‐2005	Individual	Quasi‐experimental
Dunford et al. (1982)	Kansas City	Technical report	Kansas City, Missouri, USA	1977‐1979	Individual	Random without matching
Dunford et al. (1982)	New York City	Technical report	New York City, New York, USA	1977‐1979	Individual	Random without matching
Haines et al. (2012)	Lewisham	Technical report	Lewisham, London, UK	2009‐2011	Individual	Quasi‐experimental (matched individuals)
Haines et al. (2012)	Wolverhampton	Technical report	Wolverhampton, UK	2009‐2011	Individual	Quasi‐experimental (matched individuals)
Henderson (1997)		MA thesis	Unnamed southern‐Ontario region, Canada	1994‐1997	Individual	Quasi‐experimental (control for baseline differences)
Klein (1986)		Journal	Unnamed police department area, USA	1975‐1978	Individual	Random without matching
Koch (1985)		PhD thesis	Unnamed county, Michigan, USA	1981‐1983	Individual	Random without matching
Lipsey et al. (1981)	Tie‐breaker	Journal	Los Angeles, California, USA	1974‐1977	Individual	Random without matching
Lipsey et al. (1981)	Matched group	Journal	Los Angeles, California, USA	1974‐1977	Individual	Quasi‐experimental (matched individuals)
Little (2015)		PhD thesis	Queensland, Australia	2000‐2010	Individual	Quasi‐experimental (matched individuals)
McCold & Wachtel (1998)		Technical report	Bethlehem, Pennsylvania, USA	1995‐1997	Individual	Random after matching
Sherman et al. (2000)	Shoplifting offenders	Technical report	Canberra, Australia	2000	Individual	Random without matching
Sherman et al. (2000)	Personal property crime offenders	Technical report	Canberra, Australia	2000	Individual	Random without matching
Smith, E. et al. (2004)		Journal	Unnamed city, USA	Not reported	Individual	Random without matching
Smith, P. et al. (1979)		Technical report	Long Beach, California, USA	Not reported	Individual	Random without matching
University Associates (1986)	Detroit	Technical report	Detroit, Michigan, USA	1981‐1984	Individual	Random without matching
University Associates (1986)	Kalamazoo	Technical report	Kalamazoo, Michigan, USA	1982‐1985	Individual	Random without matching

**Table 2:** Diversion characteristics and study information

**Authorship (Year)**	**Study qualifier**	**Diversion type**	**Control intervention**	**Outcome measure**	**Length of follow‐up (months)**	**Sample size**	**Gender composition**	**Ethnic composition**	**Min age**	**Max age** ^2^
** **						**Treatment/Control**	**Male**	**White/African American/Hispanic/Asian/Other** ^1^	** **	
Byles & Maurice (1979)		Caution with referral to external services	Formal court processing	Arrest or police contact	24	154/151	75‐89%	–	6	14
				Court appearance						
Cunningham (2007)		Traditional caution only	Formal court processing	Arrest or police contact	12	1232/595	50‐74%	–	10	16
		Police restorative caution				917/595				
Dunford et al. (1982)	Kansas City	Traditional caution only	Formal court processing	Arrest or police contact	6/12	95/107	75‐89%	33/67	11	17
		Caution with referral to external services (Service RFY)				100/107	75‐89%			
		Caution with referral to external services (Service YSU)				112/107	90‐99%			
Dunford et al. (1982)	New York City	Traditional caution only	Formal court processing	Arrest or police contact	6/12	193/152	90‐99%	20/47/33	11	15
		Caution with referral to external services		Arrest or police contact		180/152				
Haines et al. (2012)	Lewisham	Caution with referral to external services	Formal court processing	Court contact	15‐30	57/52	50‐74%	68/10/0/2/20	10	17
Haines et al. (2012)	Wolverhampton	Caution with referral to external services	Formal court processing	Court contact	15‐21	19/16	50‐74%	68/10/0/2/20	10	17
Henderson (1997)		Traditional caution only	Formal court processing	Court contact	24	94/70	50‐74%	90/./././.	12	17
Klein (1986)		Traditional caution only	Formal court processing	Arrest or police contact	6/15/27	82/81	–	–	–	–
				Self‐reported delinquency (frequency)	9	45/48				
				Self‐reported delinquency (severity)	9					
		Caution with referral to external services (without purchase)	Formal court processing	Arrest or police contact	6/15/27	88/81				
				Self‐reported delinquency (frequency)	9	53/48				
				Self‐reported delinquency (severity)	9					
		Caution with referral to external services (with purchase)	Formal court processing	Arrest or police contact	6/15/27	55/81				
				Self‐reported delinquency (frequency)	9	35/48				
				Self‐reported delinquency (severity)	9					
Koch (1985)		Caution with referral to external services	Formal court processing	Arrest or police contact	3.72	79/78	50‐74%	74/23/3/0/0	14.7	16
		Traditional caution only				86/78				
Lipsey et al. (1981)	Tie‐breaker RCT	Traditional caution only	Probation	Arrest or police contact	6	8/11	–	–	–	–
Lipsey et al. (1981)	Matched‐group	Traditional caution only	Probation	Arrest or police contact	6	44/118	–	–	–	–
Little (2015)		Traditional caution only	Formal court processing	Recontact with youth justice system (within two years)	24	660/660	50‐74%	–	10	16
				Recontact with youth justice system (by age 19.5 years)	Age‐dependent					
				Number of recontacts with youth justice system (within two years)	24					
				Number of recontacts with youth justice system (by age 19.5 years)	Age‐dependent					
McCold & Wachtel (1998)		Police restorative caution	Formal court processing	Arrest or police contact	6/12	189/103	50‐74%	40/8/50/0/3	–	17
Sherman et al. (2000)	Shoplifting offenders	Police restorative caution	Formal court processing	Court contact	12	73/62	50‐74%	–	–	–
Sherman et al. (2000)	Personal‐property crime offenders	Police restorative caution	Formal court processing	Court contact	12	124/115	75‐89%	–	–	–
Smith, E. et al. (2004)		Caution with referral to external services	Formal court processing	Arrest or police contact	12	137/124	75‐89%	./91/././.	14	–
		Traditional caution only				134/124				
Smith, P. et al. (1979)		Traditional caution only	Formal court processing	Arrest or police contact	6/12	29/26	90‐99%	65/29/0/0/6	–	–
		Caution with referral to internal services				22/26				
		Caution with referral to external services				29/26				
University Associates (1986)	Detroit	Traditional caution only	Formal court processing	Arrest or police contact	12	136/124	75‐89%	9/91/0/0/0	14.3	–
				Self‐reported delinquency	4/8	127/115				
		Caution with referral to external services	Formal court processing	Arrest or police contact	12	135/124				
				Self‐reported delinquency	4/8	127/115				
University Associates (1986)	Kalamazoo	Caution with referral to external services	Formal court processing	Court contact	12	164/149	50‐74%	75/22/0/0/3	14.2	–
				Self‐reported delinquency	4/8	148/131				
		Traditional caution only		Court contact	12	174/149				
				Self‐reported delinquency	4/8	146/131				

1 Ethnic composition has reported in several ways across the studies. For example, some studies only chose to describe the proportion of White or African American participants, while other counted described Hispanic and Asian participants in the ‘Other’ category. For this reason, we urge caution when interpreting these statistics.

2 Although maximum age was not reported in many studies, studies that included an adult sample, which we defined as age 18 years or over, were excluded. Therefore, the maximum possible age for all studies is 17 years.

**Table 3:** Risk of bias

**Authorship (Year)**	**Qualifier**	**Selection** ^1^	**General attrition** ^2^	**Differential attrition** ^3^	**Measure** ^4^	**Non‐reporting** ^5^
** **					**Official records/Self‐reported delinquency**	** **
Byles & Maurice (1979)		High	Low	Low	No	No
Cunningham (2007)		High	Low	Low	No	No
Dunford et al. (1982)	Kansas City	Low	Low	Low	No	No
Dunford et al. (1982)	New York City	High	Low	Low	No	No
Haines et al. (2012)	Lewisham	High	Low	Low	No	No
Haines et al. (2012)	Wolverhampton	High	Low	Low	No	No
Henderson (1997)		Unclear	Low	Low	No	No
Klein (1986)		Unclear	Low	Low	No/Yes	No
Koch (1985)		High	High	Low	No	No
Lipsey et al. (1981)	Tie‐breaker RCT	Low	Low	Low	No	No
Lipsey et al. (1981)	Matched‐group	Unclear	Low	Low	No	No
Little (2015)		Low	Low	Low	No	No
McCold & Wachtel (1998)		Low	Low	Low	No	No
Sherman et al. (2000)	Shoplifting offenders	Low	Low	Low	No	No
Sherman et al. (2000)	Personal‐property crime offenders	Low	Low	Low	No	No
Smith, E. et al. (2004)		Low	Low	Low	No	No
Smith, P. et al. (1979)		Low	Low	Low	No	No
University Associates (1986)	Detroit	Low	Low	Low	No/Yes	No
University Associates (1986)	Kalamazoo	Low	Low	Low	No/Yes	No

1 Were there meaningful differences between groups at baseline?

2 Was there a risk of general attrition bias for the primary outcome measure?

3 Was there a risk of meaningful differential attrition bias for primary outcome measure?

4 Did the measure used create a risk of bias on the part of the assessor?

5 Is there any evidence that the authors have not reported findings for all variables measured as part of this study?

**Table 4:** Analysis of Odds Ratio Effect Sizes: Means and 95% Confidence Intervals.

Analysis	*k*	Mean Odds Ratio	95% C.I.		τ^2^
			Lower	Upper	
Overall					
Selected Effect Sizes	31	0.78	0.63	0.96	0.141
All Effect Sizes	67	0.82	0.66	1.00	0.080
By Research Design					
Selected Effect Sizes					
Random Assignment	24	0.82	0.66	1.02	0.038
Quasi‐experimental	7	0.72	0.41	1.27	0.222
All Effect Sizes					
Random Assignment	55	0.87	0.72	1.05	0.037
Quasi‐experimental	12	0.73	0.40	1.34	0.241
Risk of Selection Bias					
Selected Effect Sizes					
Low Risk	17	0.81	0.74	0.88	0.000
High Risk	9	0.81	0.42	1.57	0.374
Unclear Risk	5	0.67	0.21	2.09	0.158
All Effect Sizes					
Low Risk	35	0.87	0.76	0.99	0.032
High Risk	15	0.81	0.41	1.60	0.383
Unclear Risk	17	0.68	0.23	2.06	0.140
Low Risk of Bias on All Indicators					
Selected Effect Sizes	16	0.82	0.68	0.98	0.000
All Effect Sizes	23	0.89	0.74	1.06	0.187
Type of Diversion					
Selected Effect Sizes					
Diversion only	11	0.67	0.49	0.90	0.174
Diversion plus service referral	14	0.82	0.63	1.06	0.043
Diversion plus police lead restorative justice	4	0.62	0.28	1.39	0.139
All Effect Sizes					
Diversion only	25	0.69	0.52	0.91	0.168
Diversion plus service referral	33	0.84	0.67	1.05	0.043
Diversion plus police‐led restorative justice	5	0.81	0.31	2.14	0.436
Publication Type					
Selected Effect Sizes					
Journal articles	8	0.91	0.51	1.61	0.150
Other manuscript types	23	0.73	0.58	0.92	0.125
Country of Study					
United States	22	0.76	0.63	0.91	0.009
Australia, Canada, or UK	9	0.82	0.50	1.36	0.257

Notes: Direction of odds ratio coded so that values less than 1 reflect less recidivism for the diversion group relative to the comparison group. All analyses performed in Stata using the *robumeta* command that produces robust standard errors accounting for clustering (statistical dependencies across effect sizes). *Selected Effect Sizes* analyses had one effect size per treatment‐comparison contrast, selecting the most general measure of official recidivism measured nearest 12‐months post police encounter. There were 31 such contrasts nested within 19 independent studies, across 14 publications. *All Effect Size* analyses used all available effect sizes. There were 67 effect sizes available for analysis across the 31 contrasts. The traditional homogeneity test, *Q*, is not produced by *robumeta*. However, τ^2^ provides information on heterogeneity. When τ^2^ is zero, the distribution is homogeneous. As τ^2^ increases, it indicates increasing levels of heterogeneity. Running the above analyses using David Wilson's *meanes* macro for Stata suggests that for these data, any τ^2^ above 0.05 reflects statistically significant heterogeneity.
